# Intravenous versus subcutaneous immunoglobulin in patients with haematological malignancies: time-driven activity-based costing

**DOI:** 10.1007/s00520-026-10551-y

**Published:** 2026-03-28

**Authors:** Sara Carrillo de Albornoz, Helen Haysom, Allison Mo, Jessica Guglielmino, Terri Dunstan, Kylie Rushford, Amanda Ellison, Karinna Saxby, Dan Andrew, Angelene Jesurajah, Philomina Banahene, Erin Hu, Loo Sin Hoo, Dennis Petrie, Erica M. Wood, Alisa M. Higgins, Zoe K. McQuilten

**Affiliations:** 1 Transfusion Research Unit, School of Public Health and Preventive Medicine, Monash University, Melbourne, Australia; 2 Centre for Health Economics, Monash University, Melbourne, Australia; 3 Monash Haematology, Monash Health, Melbourne, Australia; 4 Monash Pathology, Monash Health, Melbourne, Australia; 5 Medical Infusion Unit, Monash Health, Melbourne, Australia; 6 Melbourne Institute of Applied Economic and Social Research, The University of Melbourne, Melbourne, Australia; 7 Monash Pharmacy, Monash Health, Melbourne, Australia; 8 Australian and New Zealand Intensive Care Research Centre, School of Public Health and Preventive Medicine, Monash University, Melbourne, Australia

**Keywords:** Immunoglobulin, IVIg, SCIg, Haematological Malignancies, Transfusion, Cost

## Abstract

**Purpose:**

Prophylactic immunoglobulin (Ig) is used to prevent infections in patients with hypogammaglobulinaemia due to haematological malignancies (HM). Ig can be administered intravenously (IVIg) in hospital or self-administered subcutaneously (SCIg) at home, using different dosing regimens but with comparable effectiveness. In Australia, Ig product costs alone were AU$915.7 million in 2022/2023, 60% of the national blood budget. However, the total cost of IVIg and SCIg, including administration costs, remains uncertain.

**Methods:**

We conducted a prospective, time-driven, activity-based costing study to compare the costs of providing IVIg and SCIg to patients with HM from an Australian healthcare perspective. Ig product, consumables, equipment, and in-hospital costs were included. Analyses were conducted assuming full adherence and using (1) published prices for IVIg and SCIg, which excluded plasma fractionation costs to the Australian government, and (2) equivalent average weighted price for IVIg and SCIg, including plasma fractionation costs.

**Results:**

Annual IVIg product cost per patient was lower than that for SCIg under both costing scenarios: (1) AU$10,012 and (2) AU$5895, driven by higher SCIg doses. The costs of treating a patient with IVIg for a year were (1) AU$9936 and (2) AU$5787 lower than with SCIg, mainly due to higher SCIg product costs. When only in-hospital administration costs were considered (excluding Ig product and SCIg home consumables), SCIg treatment was AU$1019 less costly than IVIg.

**Conclusion:**

Our results indicated higher annual direct costs per patient treated with SCIg than IVIg, despite higher in-hospital costs associated with IVIg administration. Further research, including understanding costs to patients, is warranted.

**Supplementary Information:**

The online version contains supplementary material available at 10.1007/s00520-026-10551-y.

## Introduction

Patients with haematological malignancies (HM) are at increased risk of infections due to immunosuppression. Prophylactic immunoglobulin (Ig) is often given to these patients to prevent infections [1]. Ig is a costly plasma-derived product [2–4]  manufactured in Australia from voluntary non-remunerated plasma donations; however, this is not sufficient to meet demand and over half of all Ig is imported. In Australia, the cost of Ig is subsidised by the government via the National Blood Authority (NBA), with no cost to the patient. In 2022–2023, government expenditure towards Ig product was AU$915.7 million, accounting for 60% of the national blood budget, with hypogammaglobulinaemia secondary to HM being the leading indication [4].

The effectiveness of Ig versus no Ig on infection prevention and survival remains unclear, with supporting evidence based on trials with small patient numbers and published prior to the emergence of targeted therapies [5]. The cost-effectiveness of Ig versus no Ig is also uncertain [6]. A recent Australian trial-based economic evaluation comparing Ig (IVIg or SCIg) to prophylactic antibiotics showed Ig was more costly and did not lead to increased quality-adjusted life-year (QALY) gains [3]. This is consistent with a previous cost-effectiveness analysis comparing IVIg to no Ig in patients with chronic lymphocytic leukaemia [7].

Ig can be administered intravenously (IVIg) in-hospital, typically every 4 weeks, or self-administered subcutaneously (SCIg) at home weekly, with comparable effectiveness [1, 8]. Studies comparing IVIg and SCIg in primary immunodeficiencies have suggested SCIg may be less costly than IVIg [9–11]. However, none of these studies was conducted in the Australian setting or in patients with HM. The adoption of SCIg in Australia has been slow, with only 16% of patients receiving SCIg across eligible indications [12], and only 13% of patients with HM receiving Ig are treated with SCIg [4]. Two of the factors supporting SCIg uptake are the cost-effectiveness of SCIg versus IVIg and potential cost savings for hospitals and the health system [12]. However, the cost and cost-effectiveness of SCIg versus IVIg in patients with HM are uncertain; informed by only one Australian cost-effectiveness analysis where SCIg dominated IVIg (i.e. SCIg was more effective and less costly). However, this study had serious limitations [6], with their estimates based on unpublished data from a very small patient cohort (*n* = 13) with secondary hypogammaglobulinaemia following unspecified malignancy [8, 13]. The total costs associated with IVIg and SCIg treatment from the healthcare perspective remain unclear due to the lack of high-quality costing studies in this population [6].

Time-driven activity-based costing (TDABC) is a microcosting approach developed to accurately estimate the direct costs of treating patients across the care pathway [14], and it has been used across multiple medical conditions and healthcare settings [15, 16]. In this study, we used TDABC to determine the direct cost of providing Ig to patients with HM from a healthcare perspective, including the cost of Ig product and administration, and to compare the overall costs associated with IVIg versus SCIg.

## Materials and methods

### Study design and setting

TDABC applies two core parameters: (1) the unit cost of each resource and (2) the time required to perform an activity (multiplied by the frequency/quantity of use). Through process mapping, TDABC captures total costs associated with healthcare resources and staff time [15–17]. 

We applied previously established TDABC methods [18, 19] to estimate Ig administration costs. Data were prospectively collected at Monash Medical Centre (MMC), a tertiary university hospital in Melbourne, Australia, from January to December 2023 for all activities associated with an IVIg and SCIg episode, including staff time, resources used, and the probability of each activity occurring along the treatment pathway.

In Australia, patients with HM are eligible to receive prophylactic Ig if they present with “significant hypogammaglobulinaemia with serum IgG less than 4 g/L, or serum IgG ≥ 4 g/L with at least one life-threatening infection in the last 12 months, or serum IgG ≥ 4 g/L but less than the lower limit of the age-related reference range with at least two serious infections in the last six months requiring more than standard courses of antibiotics” [20]. IVIg is administered every 4 weeks in hospital at a typical dose of 0.4 to 0.6 g/kg of body weight and SCIg is self-administered weekly at home at a dose of 0.1 to 0.15 g/kg (although dosage can vary) [20]. Initial review by a specialist is required within 6 months and ongoing reviews at least annually to assess clinical benefit. In principle, Ig should be continued only if there is a demonstrated clinical benefit [20].

Ig is fully subsidised by the Australian government, with no direct cost to the patient. SCIg access includes hospital-based training and support, and covers the cost of consumables and equipment to support self-administration [21]. Nurse-led patient support programmes are also provided free of charge by SCIg manufacturers to support patients in home-based self-administration [22]. 

Ig dispensing data were obtained from BloodNet [23], Australia’s online system for accessing government-funded Ig products, and linked to hospital data for all patients with HM who received IVIg or SCIg in 2023 at MMC. All IVIg patients receive their infusions at the outpatient Medical Infusion Unit (MIU). SCIg patients receive their first two injections (new patients) at the MIU as part of the self-administration training led by the Monash Health SCIg nurse, after which they self-administer at home (continuing patients), with product and consumables collected from the hospital pharmacy every 8 weeks. We separated the costs by new and continuing patients given that new SCIg patients were admitted to the MIU for two trainings with the SCIg nurse on self-administration while continuing patients no longer attended the hospital to receive infusions.

### Process mapping

The processes directly related to the delivery of IVIg and SCIg treatment were mapped through staff consultation and direct observation. Every activity included in each process was identified and staff time was recorded three times (when possible) to account for variations in care. Infrequent events that were unable to be timed during the observation period were estimated by expert consensus (e.g. infusion reaction investigations).

**Fig. 1:**
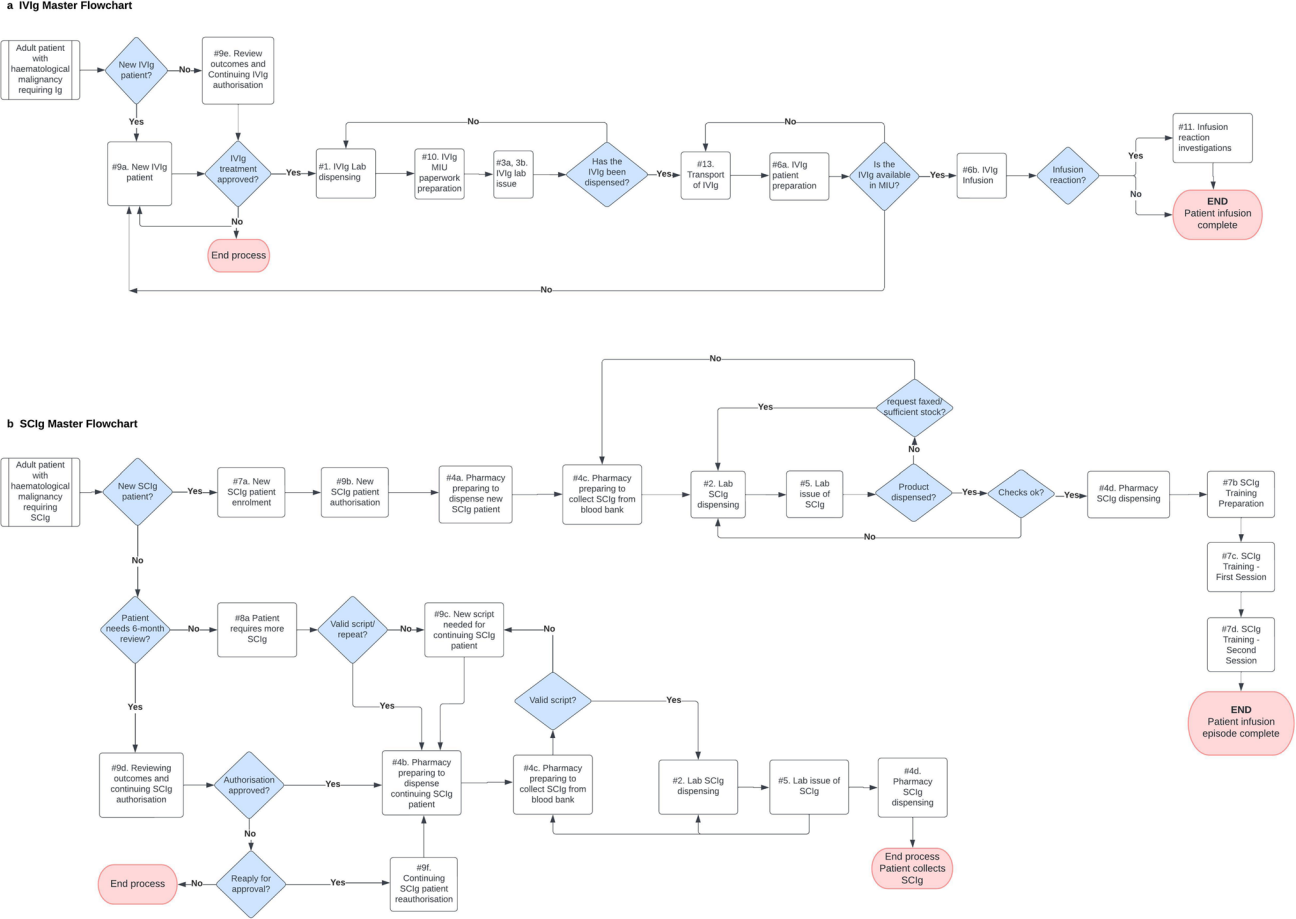
Master flowchart including all processes associated with an IVIg (**a**) and SCIg (**b**) infusion episode. Individual flowcharts for each process can be found in the supplementary materials. Processes occurring at regular intervals were not included in these flowcharts but added to the annual costs: blood fridge checks and maintenance, Ig order and delivery, SCIg patients require more consumables, SCIg administration training and competency report. Abbreviations: IVIg, intravenous immunoglobulin; lab, laboratory; MIU, medical infusion unit

Each process map included a corresponding flowchart that illustrated the potential sequence of activities within the process. The probability for different pathways occurring within the process was calculated using hospital data when available or estimated by senior staff from relevant departments (i.e. laboratory, MIU, pharmacy, haematology), who also checked all data collected and flowcharts for accuracy and consistency. In total, 32 detailed process maps were created (see supplementary materials) and combined into two separate master flowcharts: one for IVIg and one for SCIg (Fig. 1). IVIg and SCIg administrative and oversight duties that could not be timed were included as fixed overheads.

### Resource use and costs

Resource use data, including Ig and equipment utilisation, were collected from BloodNet and hospital data from January 2023 to December 2023 (Table 1). Costs for consumables and equipment used in each process were provided by a senior staff member from the relevant department. These costs were incorporated into the cost of processes where they were used (Table S4 and Table S5).

**Table 1:** Ig infusion-related inputs from Monash Health in 2023

	**IVIg**	**SCIg**
**Ig patients with HM**		
Number of patients with HM receiving Ig in 2023, *n*	60	25
Proportion of new patients, *n* (%)	31 (52.0)	17 (68.0)
Ig dose (g) per patient, mean (SD)	30.5 (9.0)	8.7 (1.3)
**Ig episodes in HM**		
Total number of Ig episodes in HM	484	669
Ig episodes per patient in 2023, mean (SD)	8.1 (4.2)	27.3 (19.8)
Ig episodes in new patients, mean (SD)	5.5 (3.4)	18.7 (15.3)
Ig episodes in continuing patients, mean (SD)	10.8 (2.8)	45.5 (15.8)
Infusion reactions reported per episode, *n*/*N* (%)	8 (0.01)	0 (0)
Number of reported severe adverse reactions, *n*/*N* (%)	2 (0.004)	0 (0)
**Ig dispensing**		
Ig total (g) in 2023 across all indications	160,400	34,102
Ig total (g) in 2023 in HM	14,915	5833
%Ig use in HM	9.3%	17.1%
% Ig imported in HM	33.3%	100%
**Ig product cost per episode**		
BloodNet Ig product cost^a^	AU$1892	AU$665
NBA Ig product cost^b^	AU$3216	AU$917
**Ig product cost per year**^**c**^		
BloodNet Ig product cost	AU$24,592	AU$34,604
NBA Ig product cost	AU$41,811	AU$47,706

Source: BloodNet and Hospital Records. Ig infusion data from BloodNet data was confirmed with hospital records. The NBA provided a weighted average price for IVIg and SCIg, including the cost of plasma fractionation. All costs are in 2023 Australian dollars

*AU$*, Australian dollars; *HM*, haematological malignancies; *Ig*, immunoglobulin; *IVIg*, intravenous immunoglobulin; *SCIg*, subcutaneous immunoglobulin; *SD*, standard deviation

^a^Ig product cost per episode was calculated from the price per gramme for each Ig product in BloodNet and mean dose (g) per episode, as a weighted average of the imported/domestic Ig. This excluded the cost of domestic plasma fractionation

^b^An average weighted product cost per gramme for Ig (same for IVIg and SCIg), including the cost of plasma fractionation to the Australian government, was provided by the NBA. Ig product cost per episode was calculated from this weighted price per gramme and mean dose per episode

^c^Assuming IVIg is administered once every 4 weeks (13 episodes per year), SCIg is self-administered once per week (52 episodes per year)

Two analyses were conducted using separate Ig product costs: (1) using BloodNet IVIg and SCIg product published prices; (2) using NBA-provided Ig product weighted average cost (same Ig cost for IVIg and SCIg). In the first scenario (1), the unit cost for Ig products was obtained from BloodNet and the cost of IVIg and SCIg product per episode was calculated as a weighted average, considering the proportion of imported and domestic products used, as well as using the mean dose per gramme per episode. Commercial-in-confidence agreements between Ig manufacturers and the NBA mean these prices may not fully reflect the cost to the healthcare system. In particular, domestic plasma-derived products are supplied by CSL Behring under the National Fractionation Agreement for Australia, but the cost of plasma fractionation to the Australian government was not included in the BloodNet product prices. This could make the cost of domestic Ig product potentially higher than that of imported Ig. The NBA report on the use of Ig highlighted there is a continuing increase in the price of plasma for fractionation due to the increased ratio of apheresis to whole blood plasma for fractionation being supplied, resulting in an increase in the cost of domestic Ig [4]. In order to address this uncertainty, a second scenario was explored (2); the NBA provided (private communication) an average weighted cost for Ig (IVIg and SCIg) of AU$105.45 per gramme (2022/2023 price), which included the cost of plasma fractionation to the Australian Government. Individual product prices for IVIg and SCIg were not provided, but the NBA communicated that “there is very little difference between the cost of IVIg and SCIg once fractionation processes are taken into consideration”.

The cost of equipment per Ig episode was calculated using equipment acquisition costs and estimated life years (i.e. expected time the equipment would be used before replacing it), the proportion of patients with HM in 2023 (to allocate equipment costs to the HM population), and the number of episodes where that equipment was used. Maintenance costs were included within the acquisition costs. Any clinical equipment related to the provision of IVIg and SCIg in hospital was included in the costs (Table S5). Non-clinical equipment and consumables (e.g. computers, printers) were not included due to the challenges associated with assigning their use to each Ig-related episode in HM and the minimal cost that would incur. Similarly, indirect hospital expenses (e.g. property, finance) were not included.

Staff salaries were obtained from published enterprise agreements and the hospital finance department and included 30% on-costs (Table S6). For laboratory, pharmacy, and MIU processes that included staff with different seniority levels, a weighted average was estimated based on rosters or expert opinion. Indirect costs to the patient (e.g. transport costs, productivity loss) were not included.

Furthermore, we conducted a sensitivity analysis to estimate the cost of an “empty infusion chair” using the indirect cost for “Pharmacotherapy for Neoplastic Disorders” (Australian Refined Diagnosis Related Group [AR-DRG] R63Z) as a proxy, as both IVIg and anticancer treatments are given as a same-day infusion in the MIU with similar duration. The indirect cost per admission episode under AR-DRG R63Z was AU$332, including all the indirect costs to the hospital associated with the intervention (e.g. clinical, non-clinical, accommodation, depreciation) and excluding on-costs (AU$31) to avoid duplication [24].

All costs were calculated in 2023 Australian dollars (AU$), with results in US$ presented in the supplementary material [25].

### Data analysis

The base-case model estimated annual costs associated with the IVIg and SCIg infusion episodes by adding up the cost of each process (see supplementary materials, Table S4 for detailed calculations), the probability of that process occurring, and assuming a full year of IVIg and SCIg with 100% adherence to treatment. The model assumed IVIg patients received 13 infusions (one every 4 weeks) and SCIg patients had 52 infusions (one every week). Processes included in the IVIg and SCIg flowcharts were incorporated into the model according to the number of times they were repeated within a year (Tables S7–S9). Processes occurring at regular intervals (e.g. fridge checks, Ig order, and delivery) were added to the final calculation. The base case included administration, Ig product costs, and SCIg home consumables. A second scenario was conducted to estimate in-hospital administration costs only, where Ig product costs and SCIg home consumables were excluded. This scenario aimed to explore in-hospital costs, as costs of SCIg home consumables are not funded by the hospital directly, but by the State and Federal Governments [21, 26].

We used bootstrapping to estimate the 95% confidence intervals around the total annual costs and cost difference between IVIg and SCIg (supplementary materials), with 1000 iterations run with random draws from bounded uniform distributions for process probabilities. We assumed no treatment switching between IVIg and SCIg.

Deterministic sensitivity analyses were also conducted (Fig. 3 and Figure S1) to explore the main cost drivers. An additional sensitivity analysis was performed, based on the utilisation observed within the study period at MMC (Table S3). As new IVIg and SCIg patients started Ig treatment at different times through the year, and thus underwent varying number of episodes per year, it was considered more appropriate to model a full year of treatment under the assumption of complete adherence to treatment as clinically indicated to ensure cost comparability.

## Results

A total of 60 and 25 patients with any HM received IVIg or SCIg, respectively, at MMC in 2023 (Table 1). The total numbers of episodes in 2023 were 484 for IVIg (8.1 episodes per patient) and 669 for SCIg (27.3 episodes per patient), of which 34 were in-hospital SCIg training sessions. The mean Ig dose per patient per infusion episode was 30.5 g for IVIg and 8.7 g for SCIg. This resulted in a lower annual mean dose per patient for IVIg (396.5 g) versus SCIg (452.4 g), based on the modelled infusion frequency of 13 versus 52 infusions per year, respectively.

The proportions of all IVIg and SCIg used in HM out of all Ig dispensed at MMC were 9.3% and 17.1%, respectively. All SCIg products were imported, compared to only 33.3% of IVIg. Overall, the Ig product costs per episode were estimated to be AU$1892 for IVIg and AU$665 for SCIg, which leads to a lower annual cost of product for IVIg compared to SCIg (AU$24,592 vs. AU$34,604), as IVIg is infused every 4 weeks (13 episodes per year) while SCIg is administered weekly (52 episodes per year). Even when assuming the same price per gramme for IVIg and SCIg, the annual product costs (including the cost of plasma fractionation) would be lower for IVIg compared to SCIg (AU$41,811 vs. AU$47,706) due to a higher mean dose in SCIg patients.

### Ig infusion-related processes and cost

The master flowcharts (Fig. 1) present the processes included in the IVIg and SCIg treatment pathways. Detailed flowcharts for each process are presented in the supplementary material.

There were key differences in the SCIg and IVIg processes. The process maps for new SCIg patients started at enrolment into the SCIg programme, while for continuing patients the SCIg episode started either at their 6-month review or when they required more SCIg and consumables. IVIg patients started either at their initial specialist consultation where IVIg was prescribed for the first time, at their 6-month specialist review, or at the laboratory IVIg dispensing process for patients receiving regular IVIg treatment. IVIg is dispensed directly by the laboratory’s blood bank to the MIU. The SCIg dispensing process has an additional step—from the blood bank to the pharmacy—before the pharmacy dispenses the product (to either the SCIg nurse for patient training or directly to the patient). Other key differences include additional steps for SCIg specialist consultations and the dedicated role of the SCIg nurse within the MMC SCIg programme.

Table 2 illustrates the cost of each process and the contribution of each process to the annual cost per patient, according to how often the process occurs in the treatment pathway (Table S1 presents the same costs in US$). Process costs alone do not reflect how often each process takes place in the treatment pathway; for example, the cost of the IVIg infusion, including IVIg patient preparation and infusion, is only AU$51.5, but over 1 year each patient is expected to undertake this process 13 times, with the total annual cost of this process amounting to $467.6 per patient. It is important to note that only direct staff time related to the infusion episode was measured, not patient time. For example, an IVIg infusion can take more than 2 h, but only the time nurses spent performing infusion-related activities was measured and, given MIU nurses alternate between different patients and tasks while the IVIg patient is receiving the infusion, the actual staff time cost was less than 2 h.

**Table 2:** IVIg and SCIg infusion processes and costs

IVIg processes	Setting	Process cost (AU$)	Cost per patient per year (AU$)^
Laboratory IVIg dispensing	Laboratory	$0.82	$12.84
Laboratory IVIg issue (individual)	Laboratory	$0.17	$0.07
Laboratory IVIg issue (multiple)	Laboratory	$0.03	$0.33
Laboratory Infusion reaction investigation	Laboratory	$40.13	$0.00
Transport of IVIg from Blood Bank to MIU	Laboratory	$0.95	$12.59
Ig order and delivery	Laboratory	$0.10	$35.07
Blood fridge checks and maintenance	Laboratory	$21.23	$0.05
New IVIg patient authorisation	Specialist	$4.96	$4.19
Reviewing outcomes and continue authorisation for IVIg	Specialist	$3.98	$9.65
IVIg MIU preparation day prior to infusion	MIU	$18.67	$247.64
IVIg infusion (from arrival at MIU to discharge)	MIU	$51.43	$676.63
**SCIg processes**	**Setting**	**Process cost (AU$)**	**Cost per patient per year (AU$)^**
Laboratory SCIg dispensing	Laboratory	$1.63	$9.91
Laboratory SCIg issue	Laboratory	$0.34	$2.24
Ig order and delivery	Laboratory	$0.10	$35.07
Blood fridge checks and maintenance	Laboratory	$21.23	$0.05
Pharmacy preparing to dispense (new) SCIg patient	Pharmacy	$0.28	$1.93
Pharmacy preparing to dispense (continuing) SCIg patient	Pharmacy	$0.24	$0.20
Pharmacy preparing to collect SCIg from blood bank	Pharmacy	$2.95	$14.97
Pharmacy SCIg dispensing	Pharmacy	$4.70	$10.73
New SCIg patient authorisation	Specialist	$18.32	$2.87
New script for continuing SCIg patient	Specialist	$2.53	$1.27
Reviewing outcomes and continue authorisation for SCIg	Specialist	$3.75	$5.67
Continuing SCIg patient re-authorisation	Specialist	$16.37	$0.98
New SCIg patient enrolment	Specialist/SCIg nurse	$6.75	$0.85
SCIg training preparation	SCIg nurse	$0.97	$0.66
First SCIg training	SCIg nurse	$59.25	$40.29
Second SCIg training	SCIg nurse	$54.24	$36.89
Patient requires more SCIg	SCIg nurse	$0.97	$5.02
Patient requires more consumables	SCIg nurse	$1.08	$0.00
SCIg Administration Training and Competency Report	SCIg nurse	$0.84	$0.00

^The process cost does not reflect how often each process occurs in the treatment pathway. The cost per patient per year accounts for how often these processes occur in a given year for an average patient, according to the proportion of new and continuing patients. Complex loops illustrated in Fig. 1 (arrows going backwards) are not included in these estimates; and therefore, the sum of these processes does not add up to the total administration costs presented in the base case results. All costs are in 2023 Australian dollars

*IVIg*, intravenous immunoglobulin; *mins*, minutes; *SCIg*, subcutaneous immunoglobulin

### TDABC results

The TDABC model (Table 3) compared the total cost of IVIg versus SCIg in a patient with HM who received a full year of treatment as prescribed, including the cost of Ig product and SCIg home consumables. Table S2 in the supplement presents these costs in US$. Ig utilisation in the base-case and administration-only model is 13 infusions/year (one every 4 weeks) for all IVIg patients and 52 infusions/year (one per week) for all SCIg patients. Only direct hospital costs are included; indirect costs to the hospital and patient are not included. All costs are in 2023 Australian dollars

**Table 3:** IVIg vs. SCIg cost per patient per year (direct costs) with 100% adherence

	**Annual IVIg** **AU$ (95% CI)**	**Annual SCIg** **AU$ (95% CI)**	Cost-difference IVIg – SCIg AU$ (95% CI)
**Scenario 1. BloodNet Ig product price**^**a**^**, in-hospital administration and SCIg home consumables**			
New patient	25,954 (25,911, 26,006)	36,007 (36,005, 36,009)	−10,053 (−10,096, −10,001)
Continuing patient	25,953 (25,905, 26,000)	35,640 (35,638, 35,641)	−9687 (−9735, −9639)
Weighted average	25,953 (25,908, 26,003)	35,889 (35,888, 35,891)	−9936 (−9982, −9886)
**Scenario 2. NBA Ig product price**^**b**^**, in-hospital administration and SCIg home consumables**			
New patient	43,202 (43,124, 43,220)	49,107 (49,107, 49,111)	−5904 (−5984, −5889)
Continuing patient	43,202 (43,124, 43,219)	48,740 (48,740, 48,743)	−5538 (−5618, −5522)
Weighted average	43,202 (43,124, 43,219)	48,989 (48,989, 48,993)	−5787 (−5867, −5771)
**In-hospital administration only: excluding Ig product and SCIg home consumables**			
New patient	1362 (1319, 1414)	385 (383, 387)	977 (934, 1029)
Continuing patient	1361 (1313, 1408)	252 (251, 254)	1109 (1060, 1156)
Weighted average	1361 (1315, 1412)	342 (341, 344)	1019 (973, 1069)

^a^BloodNet prices included the price for each Ig product, but excluded the price of domestic plasma fractionation. Under this scenario, SCIg product costs were higher than IVIg due to a higher proportion of (more expensive) imported product used

^b^Average weighted product cost for IVIg and SCIg provided by the NBA, including the cost of plasma fractionation to the Australian government

*CI*, confidence interval; *IVIg*, intravenous immunoglobulin; *NBA*, National Blood Authority; *SCIg*, subcutaneous immunoglobulin

**Fig. 2:**
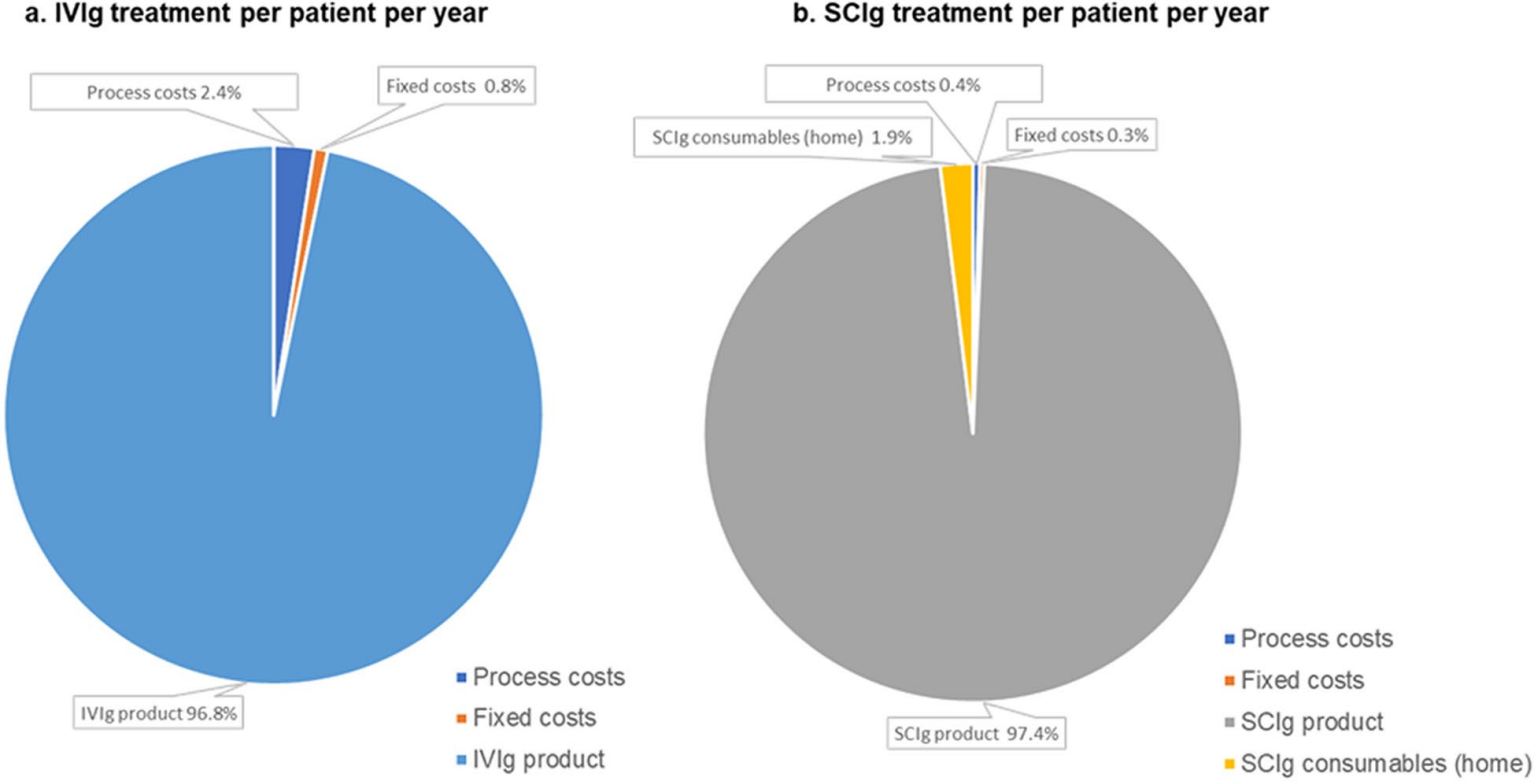
Proportion of total annual costs attributable to variable processes, fixed costs, and Ig product (assuming the same IVIg and SCIg product cost, including the cost of plasma fractionation): **a** IVIg treatment per patient per year; **b** SCIg treatment per patient per year. Based on the base-case model results in Table 3; Ig utilisations in the base-case and administration-only models are 13 infusions/year (one every 4 weeks) for all IVIg patients and 52 infusions/year (one per week) for all SCIg patients. Fixed costs include staff oversight and administration duties. Abbreviations: IVIg, intravenous immunoglobulin; SCIg, subcutaneous immunoglobulin

In the first scenario using BloodNet published costs, the first year of a new patient receiving IVIg was AU$25,954 and subsequent years were AU$25,953 for continuing patients. The corresponding annual costs for SCIg patients were AU$36,007 and AU$35,640. Using the proportion of new patients observed among IVIg (52%) and SCIg (68%) patients at MMC, the annual weighted costs per patient were AU$25,953 for IVIg and AU$35,889 for SCIg, with an annual cost difference between IVIg and SCIg of AU$9936 per patient in favour of IVIg. In the second scenario, using unpublished costs provided by the NBA including the cost for plasma fractionation, IVIg annual costs remained the same for starting and continuing patients at AU$43,202, while SCIg annual costs were slightly higher in the first year, at AU$49,107 and declined in existing patients to AU$48,740 per patient per year. The weighted average cost difference was AU$5787 per patient per year in favour of IVIg. Ig product costs were the main driver of the total annual costs, accounting for 96.8% and 97.4% of the annual costs per patient per year receiving IVIg or SCIg, respectively, when assuming equal pricing (Fig. 2). The total annual costs per patient of in-hospital Ig administration (excluding Ig product costs, SCIg home consumables, and pump) were AU$1361 for IVIg and AU$342 for SCIg, with an annual cost difference between IVIg and SCIg of AU$1019.

### Sensitivity analyses

**Fig. 3:**
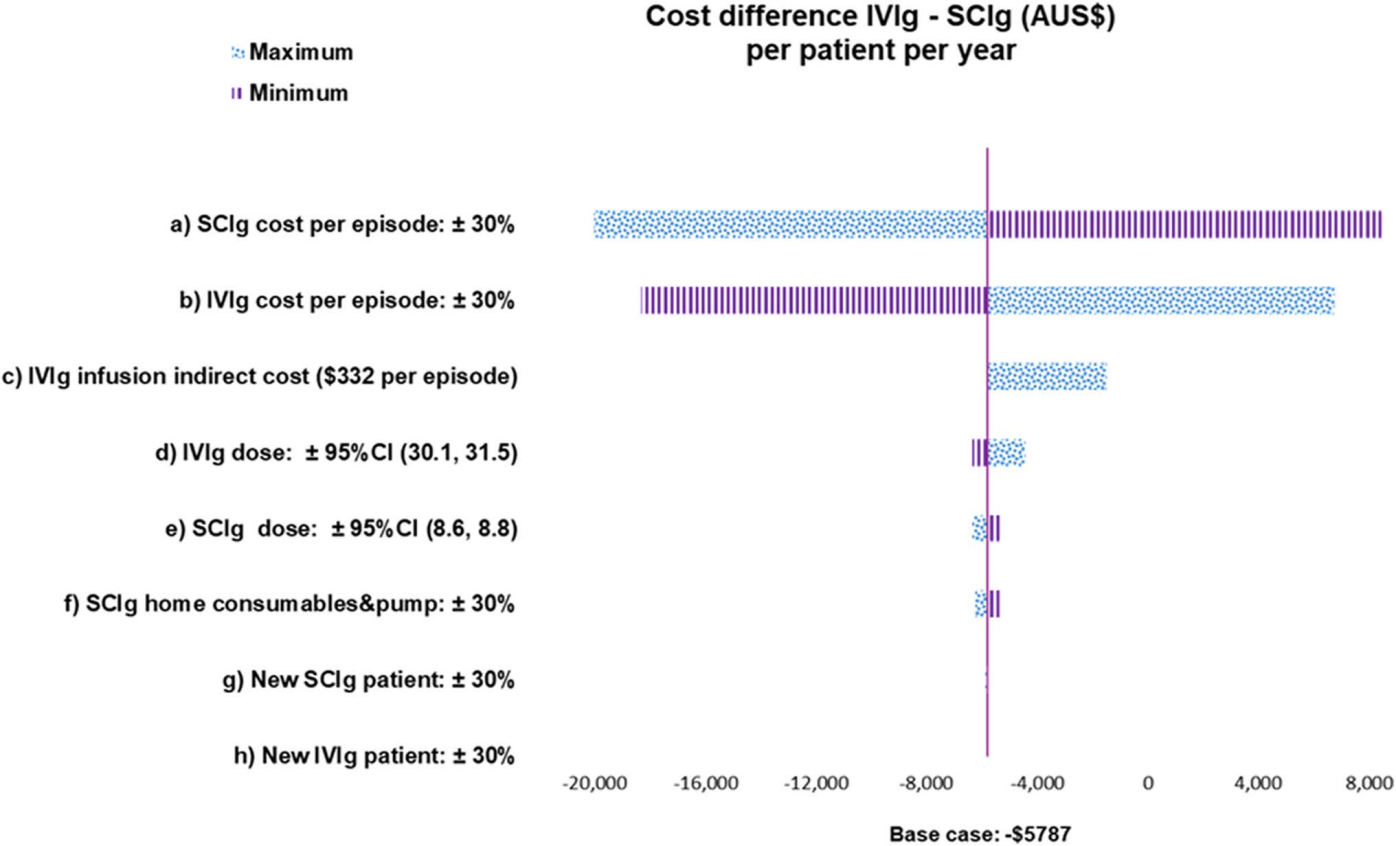
Tornado diagram deterministic sensitivity analysis on the second scenario (same product cost for IVIg and SCIg, including cost of plasma fractionation). The vertical line represents the cost difference on the annual cost per patient per year for a patient receiving IVIg vs. SCIg; i.e. higher than 0 = IVIg is more expensive, lower than 0 = SCIg is more expensive. The red bars indicate the change in the cost difference if the parameter is increased and the blue bars indicate the change in the cost difference if the parameter is decreased. All costs are in 2023 Australian dollars. Abbreviations: IVIg, intravenous immunoglobulin; SCIg, subcutaneous immunoglobulin

Deterministic sensitivity analyses were conducted by varying key parameters considered to have the greatest impact on the model (Fig. 3). We used Ig product costs from the second scenario, where IVIg and SCIg product prices per gram were assumed equal (including the cost of plasma fractionation), as this was the most conservative model. The tornado diagram for the first scenario, using BloodNet IVIg and SCIg prices, is presented in the supplement (Figure S1).

The main model driver was the cost of SCIg products; the cost difference increased to AU$20,071 in favour of IVIg when SCIg product costs were raised by 30%, while decreasing SCIg product cost by 30% made the annual cost of SCIg AU$8540 cheaper than IVIg. When considering the estimated indirect costs to the hospital associated with an IVIg infusion (i.e. not directly related to patient care), the difference between IVIg and SCIg was reduced to $1471, still in favour of IVIg. Ig dose alone, SCIg consumables and pump, and the variation on the proportion of new versus continuing patients did not have a significant impact on the cost difference.

Further sensitivity analyses assessed the impact of observed Ig utilisation patterns at MMC in 2023 (Table S3). New IVIg patients received on average six infusions and continuing patients 11 infusions per year, while new SCIg patients had 19 infusions and continuing SCIg patients had 46 infusions per year. As we measured a calendar year (2023), patients started Ig at different times and therefore had different numbers of infusions per year. Assuming the same cost for IVIg and SCIg (NBA cost), the cost difference between IVIg and SCIg for new patients was AU$1779 in favour of SCIg, and the corresponding difference for continuing patients was AU$6544 in favour of IVIg.

## Discussion

This is the first study to use TDABC methods to measure the cost of IVIg and SCIg treatment and understand the costs related to providing Ig. Our results indicated higher annual direct costs per patient treated with SCIg than IVIg, for both new and existing patients, despite higher hospital costs associated with IVIg administration. Our microcosting analysis confirms the costs of Ig administration are comparatively small, as more than 95% of the total costs were driven by Ig product costs. The cost of Ig product has been identified as a main driver in cost minimisation analyses of SCIg versus IVIg in patients with primary immunodeficiencies conducted in Germany [10], Switzerland [9], and Spain [27].

We included processes from all departments and activities that contributed to the infusion episode and continuous treatment of HM patients over 1 year. In both scenarios considered in the analysis, the main cost driver was the annual cost of SCIg product; using BloodNet published prices, the cost per gramme was higher for SCIg than IVIg, due to a higher proportion of (more expensive) imported product and higher mean dose. However, annual product costs per gramme continued to be higher for SCIg even when the same price was assumed for both IVIg and SCIg, including the cost of plasma fractionation, due to a higher mean dose in SCIg patients compared to IVIg patients. The higher SCIg dose is consistent with previous studies in HM [13, 28, 29]. It is possible SCIg doses were rounded up to minimise vial wastage or adjusted to minimise infection risk [20, 30]. 

These results may inform negotiations regarding SCIg pricing. In European studies, the cost of SCIg per gramme was reported to be lower than that of IVIg; at €84.60 vs. €38.54 per gramme in Germany [10] and at 75.85 vs. 73.05 Swiss franc per gramme in Switzerland [9]. Similarly, the annual product cost per patient was lower for SCIg in the Spanish study (€13,531.39 SCIg vs. €15,458.86 IVIg)[27]. Under the current pricing agreements, an increase in the uptake of SCIg would have different implications for individual hospitals, with lower administration costs and fewer patients in need of an infusion chair, but the Australian Department of Health, through the NBA, as the payer for Ig product may incur higher costs. Overall, there would be limited savings to the public health system from the IVIg to SCIg transition without product price changes. Even slightly higher SCIg prices compared to IVIg would result in annual cost differences favouring IVIg.

These cost minimisation studies also included indirect costs to patients and carers [9, 10, 27], such as sick leave for caregivers, lost productivity, and transport costs. Our study was conducted from the perspective of the healthcare system and only included direct hospital costs associated with the provision of Ig. The inclusion of indirect costs may narrow the cost difference between the two treatment modes, as SCIg patients avoid the need for monthly hospital visits to receive infusions and associated costs. The exclusion of indirect costs to the patient may understate SCIg’s societal value despite higher product costs. We included 30% on-costs added to the salaries to reflect leave provisions, tax, and superannuation (i.e. compulsory retirement savings system in Australia). Hospital fixed costs such as buildings, electricity, and other departments not directly involved in the infusion episode (e.g. finance, human resources) were not included. However, the opportunity cost of an “empty infusion chair” freed by a SCIg patient self-administering at home was explored under the more conservative second scenario (assuming the same product cost for IVIg and SCIg) by adding the estimated indirect cost of an IVIg infusion episode. This led to a reduction in the annual cost difference, but SCIg treatment remained more expensive than IVIg.

A limitation of our study was that it was based at a single hospital, and processes may differ at other sites, particularly in rural and remote areas. The timing of each activity was only captured three times, following the methodology in previously published studies [18]. The variability between the repeated measurements was minimal (< 10%), and given the small cost contribution of in-hospital processes compared to Ig products, these potential variations would have little impact on the total cost of Ig.

Variations in adherence may also impact total costs, as patients may use less Ig in a year than the ideal modelled scenarios. A study in patients following stem cell transplant reported worse adherence in patients treated with SCIg compared to IVIg [31], but patient numbers were small and generalisability limited. We explored the impact of lower adherence on the total costs based on annual Ig utilisation data at MMC. The cost difference between IVIg and SCIg was reversed for new and existing patients, favouring SCIg for new patients and IVIg for existing patients. These results illustrate how variations in Ig use may impact costs, but are not comparable as patients started Ig treatment at different times through the year and had different numbers of treatment episodes.

The cost of SCIg patient-support programmes [22], currently subsidised by pharmaceutical companies, could not be assessed as these are commercially confidential and held by SCIg manufacturers. Moreover, some consumables are currently given to patients free of charge by SCIg manufacturers. Changes in any of these activities by pharmaceutical companies could represent a financial risk to the public healthcare system. Lastly, we included the costs of infusion reaction investigations in the laboratory and medications given in the MIU, but the cost of subsequent emergency admissions or long-term costs associated with infusion reaction complications were not included. However, given the low proportion of severe infusion reactions in our study, it is unlikely these costs would have a significant impact. Similarly, we did not include any costs for admissions unrelated to the infusion episode (e.g. infections or admissions to receive chemotherapy).

The main strength of TDABC is to capture true resource use and associated costs—assuming equal utilisation of IVIg and SCIg consistent with the clinical indication—and to provide key insights into the treatment pathway, enabling the identification of the main cost drivers. Our TDABC study presents detailed data that allows others to compare Ig administration processes and costs across centres and health systems, and might enable multidisciplinary collaboration to optimise healthcare delivery and redesign. Furthermore, these results are particularly important for policy makers, as they can inform reimbursement decisions and support evidence-based pricing negotiations with manufacturers. Despite the potential for hospital-level cost savings associated with SCIg use, the higher SCIg annual product costs compared to IVIg mean that only a reduction in the price of SCIg would make it less costly than IVIg from a healthcare perspective.

## Supplementary Information

Below is the link to the electronic supplementary material.

ESM 1 (DOCX 6.29 MB)

## Data Availability

Data are available upon reasonable request from the corresponding author.
