# Supplementary material for: Intravenous versus subcutaneous immunoglobulin in patients with haematological malignancies: time-driven activity-based costing
Source: Support Care Cancer. 2026 Mar 28;34(4):382. doi: 10.1007/s00520-026-10551-y (PMC13032948; doi:10.1007/s00520-026-10551-y)

**SUPPLEMENTARY INFORMATION**

**Title: Intravenous versus subcutaneous immunoglobulin in patients with haematological malignancies: time-driven activity-based costing**

This appendix has been provided by the authors to give readers additional information about their work.

Supplement to: Intravenous versus subcutaneous immunoglobulin in patients with haematological malignancies: time-driven activity-based costing. Carrillo de Albornoz et al.

Authors: Sara Carrillo de Albornoz, Helen Haysom, Allison Mo, Jess Guglielmino, Terri Dunstan, Kylie Rushford, Amanda Ellison, Karinna Saxby, Dan Andrew, Angelene Jesurajah, Erin Hu, Philomina Banahene, Loo Sin Hoo, Dennis Petrie, Erica M Wood, Alisa M Higgins, Zoe K McQuilten

Corresponding author:

Sara Carrillo de Albornoz

School of Public Health and Preventive Medicine, Monash University

553 St Kilda Rd, Melbourne VIC 3004

sara.carrillodealbornoz@monash.edu

+61 3 99030219

ORCID: 0000-0002-1206-5392

[**SUPPLEMENTARY RESULTS** 4](#_Toc220487924)

[TDABC model results (US$) 4](#_Toc220487925)

[Sensitivity analyses 5](#_Toc220487926)

[Tornado diagram using the first scenario: BloodNet IVIg and SCIg prices 5](#_Toc220487927)

[Ig utilization patterns at MMC in 2023 6](#_Toc220487928)

[**SUPPLEMENTARY METHODS** 8](#_Toc220487929)

[Equipment and consumables costs 8](#_Toc220487930)

[Staffing Costs 10](#_Toc220487931)

[Calculation of process costs 11](#_Toc220487932)

[Calculation of total annual costs per patient 12](#_Toc220487933)

[Bootstrapping and 95% CI 14](#_Toc220487934)

[Individual process flowcharts 15](#_Toc220487935)

[1. Laboratory IVIg dispensing 15](#_Toc220487936)

[2. Laboratory SCIg dispensing 15](#_Toc220487937)

[3. Laboratory IVIg issue: 3a. Individual issue. 3b. Multiple issue 16](#_Toc220487938)

[4. Pharmacy SCIg dispensing: 4a. Pharmacy preparing to dispense (new) SCIg patient. 4b. Pharmacy preparing to dispense (continuing) SCIg patient. 4c. Pharmacy preparing to collect SCIg from Blood Bank. 4d. Pharmacy SCIg dispensing. 17](#_Toc220487939)

[5. Laboratory SCIg issue 18](#_Toc220487940)

[6. Admin of IVIg: 6a. IVIg patient preparation / 6b. IVIg infusion 19](#_Toc220487941)

[7. 7a. New SCIg patient enrolment. 7b. SCIg training preparation. 7c. First SCIg training. 7d. Second SCIg training 20](#_Toc220487942)

[8. 8a. Patient requires more SCIg. 8b/8c. Patient requires more consumables. 8d. SCIg Administration Training and Competency Report 21](#_Toc220487943)

[9. 9a. New IVIg patient authorisation. 9e Reviewing outcomes and continue authorisation for IVIg 22](#_Toc220487944)

[9. 9b. New SCIg patient authorisation. 9c. New script for existing SCIg patient. 9d Reviewing outcomes and continue authorisation for SCIg. 9f. Existing SCIg patient re-authorisation 23](#_Toc220487945)

[10. IVIg MIU preparation day prior to infusion 24](#_Toc220487946)

[11. Infusion reaction investigation: 11a. Laboratory testing. 11b. Elution. 11c. Analysis and notification 25](#_Toc220487947)

[12. Blood fridge checks and maintenance 26](#_Toc220487948)

[13. Transport of IVIg from Blood Bank to MIU 26](#_Toc220487949)

[14 . Ig order and delivery: 14a. Daily routine order of Ig. 14b. Daily Ig delivery 27](#_Toc220487950)

**List of Tables**

[Table S1. IVIg and SCIg infusion processes and costs (US$) 4](#_Toc207110940)

[Table S2. TDABC results: IVIg vs. SCIg cost per patient per year (direct costs US$) 5](#_Toc207110941)

[Table S3. IVIg vs. SCIg cost per patient per year (AU$ and US$) 5](#_Toc207110942)

[Table S4. Consumables Costs Associated with Ig infusion episodes in haematological malignancies 6](#_Toc207110943)

[Table S5. Equipment Costs Associated with Ig infusion episodes in haematological malignancies) 7](#_Toc207110944)

[Table S6. Staff type and the hourly wages 8](#_Toc207110945)

[Table S7. Total cost calculation for a process (example based on the process flowchart above) 9](#_Toc207110946)

[Table S8. Processes in IVIg master flowchart branches by number of times repeated within a year 11](#_Toc207110947)

[Table S9. Processes in SCIg master flowchart branches by number of times repeated within a year 11](#_Toc207110948)

# **SUPPLEMENTARY RESULTS**

## TDABC model results (US$)

**Table S1. IVIg and SCIg infusion processes and costs (US$)**

| IVIg Processes | Setting | Process cost (US$) | Cost per patient per year (US$)^ |
| --- | --- | --- | --- |
| Laboratory IVIg dispensing | Laboratory | $0.6 | $8.9 |
| Laboratory IVIg issue (individual) | Laboratory | $0.1 | $0.0 |
| Laboratory IVIg issue (multiple) | Laboratory | $0.0 | $0.2 |
| Laboratory Infusion reaction investigation | Laboratory | $0.5 | $0.0 |
| Transport of IVIg from Blood Bank to MIU | Laboratory | $15.0 | $8.7 |
| Ig order and delivery | Laboratory | $12.2 | $26.3 |
| Blood fridge checks and maintenance | Laboratory | $27.7 | $0.0 |
| New IVIg patient authorisation | Specialist | $3.4 | $2.9 |
| Reviewing outcomes and continue authorisation for IVIg | Specialist | $2.7 | $6.7 |
| IVIg MIU preparation day prior to infusion | MIU | $12.9 | $171.1 |
| IVIg patient preparation | MIU | $21.3 | $282.8 |
| IVIg infusion | MIU | $14.2 | $184.8 |
| SCIg Processes | **Setting** | **Process cost (US$)** | **Cost per patient per year (US$)^** |
| Laboratory SCIg dispensing | Laboratory | $1.12 | $6.85 |
| Laboratory SCIg issue | Laboratory | $0.23 | $1.55 |
| Ig order and delivery | Laboratory | $0.07 | $24.23 |
| Blood fridge checks and maintenance | Laboratory | $14.67 | $0.03 |
| Pharmacy preparing to dispense (new) SCIg patient | Pharmacy | $0.20 | $1.33 |
| Pharmacy preparing to dispense (continuing) SCIg patient | Pharmacy | $0.17 | $0.14 |
| Pharmacy preparing to collect SCIg from Blood Bank | Pharmacy | $2.04 | $10.35 |
| Pharmacy SCIg dispensing | Pharmacy | $3.25 | $7.42 |
| New SCIg patient authorisation | Specialist | $12.66 | $1.99 |
| New script for continuing SCIg patient | Specialist | $1.75 | $0.88 |
| Reviewing outcomes and continue authorisation for SCIg | Specialist | $2.59 | $3.92 |
| Continuing SCIg patient re-authorisation | Specialist | $11.31 | $0.68 |
| New SCIg patient enrolment | Specialist/SCIg nurse | $4.67 | $0.59 |
| SCIg training preparation | SCIg nurse | $0.67 | $0.45 |
| First SCIg training | SCIg nurse | $40.95 | $27.84 |
| Second SCIg training | SCIg nurse | $37.49 | $25.49 |
| Patient requires more SCIg | SCIg nurse | $0.67 | $3.47 |
| Patient requires more consumables | SCIg nurse | $0.75 | $4.22 |
| SCIg Administration Training and Competency Report | SCIg nurse | $0.53 | $0.36 |

^The process cost does not reflect how often each process occurs in the treatment pathway. The cost per patient per year accounts for how often these processes occur in a given year for an average patient, according to the proportion of new and continuing patients. Complex loops illustrated in Figure 1 (arrows going backwards) are not included in these estimates and, therefore, the sum of these processes does not add up to the total administration costs presented in the base case results.

Costs are in 2023 Australian dollars. US dollars were converted from AU$ to US dollars using the EPPI-Centre cost converter.

Abbreviations: IVIg: intravenous immunoglobulin; mins: minutes; SCIg: subcutaneous immunoglobulin.

**Table S2. TDABC results: IVIg vs. SCIg cost per patient per year (direct costs US$) with 100% adherence**

|  | **Annual IVIg**  **US$ (95% CI)** | **Annual SCIg**  **US$(95% CI)** | **Cost-difference IVIg – SCIg US$ (95% CI)** |
| --- | --- | --- | --- |
| **Scenario 1. BloodNet Ig product price^a^, in-hospital administration and SCIg home consumables** | | | |
| New patient | 17,940 (17,906, 17,972) | 24,884 (24,882, 24,885) | -6,944 (-6,977, -6,911) |
| Continuing patient | 17,936 (17,902, 17,968) | 24,630 (24,629, 24,631) | -6,694 (-6,728, -6,662) |
| Weighted average | 17,938 (17,904, 17,970) | 24,803 (24,801, 24,804) | -6,865 (-6,898, -6,832) |
| **Scenario 2. NBA Ig product price^b^, in-hospital administration, and SCIg home consumables** | | | |
| New patient | 29,856 (29,802, 29,868) | 33,937 (33,937, 33,939) | -4,080 (-4,136, -4,070) |
| Continuing patient | 29,856 (29,802, 29,868) | 33,683 (33,683, 33,685) | -3,827 (-3,882, -3,816) |
| Weighted average | 29,856 (29,802, 29,868) | 33,855 (33,855, 33,858) | -3,999 (-4,055, -3,989) |
| **In-hospital administration only: Ig adherence 100%, excluding Ig product and SCIg home consumables** | | | |
| New patient | 944 (911, 977) | 266 (265, 267) | 679 (645, 711) |
| Continuing patient | 940 (907, 973) | 174 (173, 176) | 766 (733, 799) |
| Weighted average | 943 (909, 976) | 237 (235, 238) | 706 (673, 739) |

Ig utilisation in the base-case and administration only model is 13 infusions/year (one every 4 weeks) for all IVIg patients and 52 infusions/year (one per week) for all SCIg patients. Only direct hospital costs are included, indirect costs to the hospital and patient are not included.

^a^ BloodNet prices included the price for each Ig product, but excluded the price of domestic plasma fractionation. Under this scenario, SCIg product costs were higher than IVIg due to a higher proportion of (more expensive) imported product used.

^b^ Average weighted product cost for IVIg and SCIg provided by the NBA, including the cost of plasma fractionation to the Australian Government.

All costs are in US dollars (2023), converted from AU$ to US dollars using the EPPI-Centre cost converter

Abbreviations: CI: confidence interval; IVIg: intravenous immunoglobulin; NBA: National Blood Authority; SCIg: subcutaneous immunoglobulin

## Sensitivity analyses

### Tornado diagram using the first scenario: BloodNet IVIg and SCIg prices

Deterministic sensitivity analyses were conducted by varying key parameters considered to have the greatest impact on the model. Figure S1 presents the tornado diagram using IVIg and SCIg costs obtained from BloodNet.

The cost per gram was made equal for SCIg and IVIg by varying the proportion of imported vs. domestic product. When imported SCIg was set to 33% (matching IVIg), the per-gram cost equalized and the annual cost difference fell to –AU$3,391. Assuming 100% imported IVIg (matching SCIg pricing) produced a cost difference of –AU$4,200.


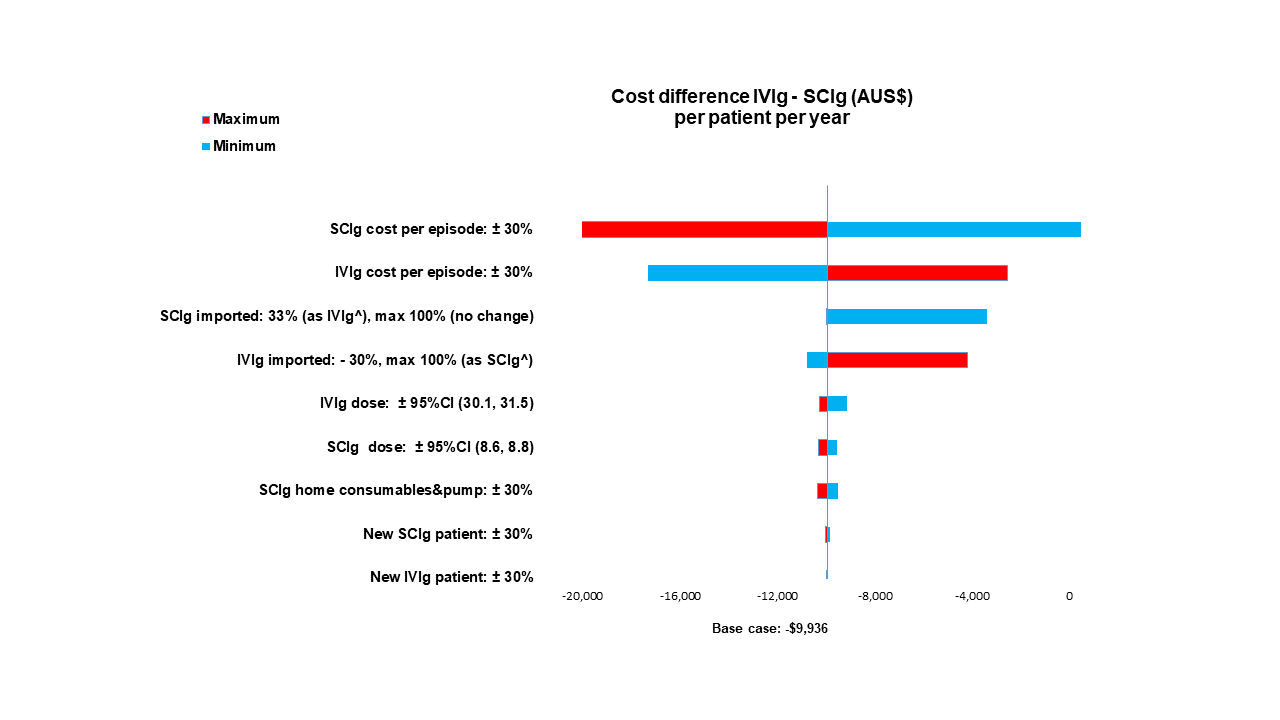


**Figure S1. Tornado diagram deterministic sensitivity analysis (scenario 1) using BloodNet Ig prices.**The vertical line represents the cost difference on the annual cost per patient per year for a patient receiving IVIg versus SCIg; i.e., higher than zero = IVIg is more expensive, lower than zero = SCIg is more expensive. The red bars indicate the change in the cost difference if the parameter is increased and the blue bars indicate the change in the cost difference if the parameter is decreased. All costs are in 2023 Australian dollars.

Abbreviations: IVIg: intravenous immunoglobulin; SCIg: subcutaneous immunoglobulin.

### Ig utilization patterns at MMC in 2023

Table S3 presents the observed Ig utilisation at MMC during the study, highlighting the impact on annual costs of observed adherence to Ig in existing patients and new patients starting at different points and not receiving a full year of treatment. We used the mean number of episodes per patient per year obtained from patients with haematological malignancies at MMC in 2023 (Table 1). New IVIg patients received 6 infusions and continuing patients 11 infusions. Among SCIg patients, new patients had 17 infusions and continuing patients had 47 infusions in 2023.

**Table S3. IVIg vs. SCIg cost per patient per year (AU$ and US$) using BloodNet Ig prices**

|  | **Annual IVIg Cost (95% CI)** | **Annual SCIg Cost (95% CI)** | **Cost difference (95% CI)** |
| --- | --- | --- | --- |
| **Ig utilization as observed at MMC (2023), including Ig product and consumables (AU$)** | | | |
| New patient | 11,999 (11,993, 12,053) | 13,395 (13,395, 13,397) | -1,396 (-1,402, -1,342) |
| Existing patient | 21,984 (21,973, 22,084) | 31,535 (31,533, 31,536) | -9,551 (-9,562, -9,451) |
| Weighted average | 16,792 (16,783, 16,868) | 19,200 (19,199, 19,201) | -2,408 (-2,416, -2,332) |
| **Ig utilization as observed at MMC (2023), including Ig product and consumables (US$)** | | | |
| New patient | 8,292 (8,288, 8,330) | 9,257 (9,257, 9,258) | -965 (-969, -927) |
| Existing patient | 15,192 (15,185, 15,261) | 21,793 (21,792, 21,794) | -6,601 (-6,608, -6,531) |
| Weighted average | 11,604 (11,599, 11,657) | 13,269 (13,268, 13,270) | -1,664 (-1,670, -1,611) |

New IVIg patients had 6 infusions/year and existing IVIg patients had 11 infusions/year; new SCIg patients had 17 infusions/year and existing SCIg patients had 47 infusions/year. BloodNet prices included the price for each Ig product, but excluded the price of domestic plasma fractionation. Under this scenario, SCIg product costs were higher than IVIg due to a higher proportion of (more expensive) imported product used. Costs are in 2023 Australian dollars. US dollars were converted from AU$ to US dollars using the EPPI-Centre cost converter.

Abbreviations: IVIg: intravenous immunoglobulin; MMC: Monash Health Medical Centre; SCIg: subcutaneous immunoglobulin

# **SUPPLEMENTARY METHODS**

## Equipment and consumables costs

Costs of consumables and equipment were provided by Monash Medical Centre (Table S4-S5). Maintenance costs were included in the acquisition costs. The total annual cost for having a particular piece of equipment was evaluated by ascertaining what percentage of the annual cost was attributable to patients with haematological malignancies. The cost per Ig episode was then calculated by the number of Ig episodes per year in these patients. The equipment costs are shown in Table S2 below.

**Table S4. Consumables Costs Associated with Ig infusion episodes in haematological malignancies**

| **Consumables** | **Cost (AU$)** | **Cost (US$)** |
| --- | --- | --- |
| IV cannula insertion pack | $3.55 | $2.45 |
| Normal Saline 0.9% 100ml | $2.68 | $1.85 |
| 22g needle | $1.63 | $1.12 |
| 10 mL syringe | $0.09 | $0.06 |
| Sterile gloves | $0.07 | $0.05 |
| Dressing | $0.23 | $0.16 |
| Handwash | $0.03 | $0.02 |
| Alcohol wipe | $0.01 | $0.01 |
| IV kit | $10.00 | $6.91 |
| Cotton ball | $0.01 | $0.01 |
| DiaCidel kit | $11.30 | $7.81 |
| Glass tubes | $0.11 | $0.07 |
| Container for diluted wash solution | $1.24 | $0.86 |
| Plastic transfer pipettes | $0.29 | $0.20 |
| Pipette tips | $0.02 | $0.01 |
| Infusion reaction medications | $1.30 | $17.94 |
| Premedications IVIg | $2.83 | $1.96 |
| Premedications SCIg | $0.01 | $0.01 |
| Needle set | $9.88 | $6.83 |
| Vented spike | $0.84 | $0.58 |
| Band-aid dot | $0.10 | $0.07 |
| Syringe hypodermic | $1.10 | $0.76 |
| Thermometer probe cover | $0.07 | $0.05 |
| Tourniquet | $0.79 | $0.55 |
| Surface wipes | $0.43 | $0.29 |
| Consumables CSL | $125.54 | $86.76 |
| Consumables Takeda | $87.84 | $60.71 |
| SCIg pump to take home^ | $235.00 | $162.40 |

^ Cost of the pump for the patient to self-infuse at home is calculated as a weighted average of the SCIg60 Infusor and Spring Infusor pump. This is costed as a consumable as it is given to the patient to take home (one-off cost), not reused by the hospital.

Costs are in 2023 Australian dollars. US dollars were converted from AU$ to US dollars using the EPPI-Centre cost converter.

**Table S5. Equipment Costs Associated with Ig infusion episodes in haematological malignancies)**

| **Equipment** | **Acquisition cost (AU$)** | **Lifetime Years** | **Cost per year (AU$)** | **Proportion used in HM** | **Cost per year in HM patients (AU$)** | **Cost per Ig infusion episode in HM patients** | |
| --- | --- | --- | --- | --- | --- | --- | --- |
|  |  |  |  |  |  | **AU$** | **US$** |
| Blood bank fridge (IVIg) | $16,000.00 | 10 | $1,600 | 0.08803 | $140.52 | $0.291 | $0.201 |
| Blood bank fridge (SCIg) | $16,000.00 | 10 | $1,600 | 0.00477 | $7.63 | $0.072 | $0.050 |
| Blood bank fridge (all Ig) | $16,000.00 | 10 | $1,600 | 0.08866 | $141.85 | $0.240 | $0.166 |
| IV pump | $750.00 | 5 | $150 | 0.04359 | $6.54 | $0.014 | $0.009 |
| IV stand | $200.00 | 3 | $67 | 0.04359 | $2.91 | $0.006 | $0.004 |
| Biovue centrifuge | $5,000.00 | 13 | $385 | 0.00003 | $0.01 | $0.000 | $0.000 |
| VisionMax | $1000 per year | 7 | $1,000 | 0.00003 | $0.03 | $0.000 | $0.000 |
| Maggylamp | $400.00 | 15 | $27 | 0.00003 | $0.00 | $0.000 | $0.000 |
| Volumetric pipette | $900.00 | 10 | $90 | 0.00003 | $0.00 | $0.000 | $0.000 |
| SCIg pump^ (in-hospital trainings) | $460.00 | 5 | $92 | 0.17105 | $3.93 | $0.236 | $0.163 |
|  | $160.00 | 5 | $32 | 0.17105 | $4.11 |  |  |
| Thermometer SCIg | $350.00 | 4 | $88 | 0.00306 | $0.27 | $0.008 | $0.005 |
| Thermometer IVIg | $350.00 | 4 | $88 | 0.04359 | $3.81 | $0.008 | $0.005 |
| Vital signs monitor SCIg | $4,895.60 | 5 | $979 | 0.00306 | $3.00 | $0.088 | $0.061 |
| Vital signs monitor IVIg | $4,895.60 | 5 | $979 | 0.04359 | $42.68 | $0.088 | $0.061 |

The cost of equipment per year was calculated by dividing equipment acquisition costs by estimated life years; the cost per year in HM patients was calculated by multiplying the cost per year by the proportional use in patients with HM; the cost per episode was calculated by dividing the cost per year in HM patients by the number of episodes in patients with HM per year.

Ig episode was calculated using, the proportion of patients with HM in 2023, and the number of episodes where that equipment was used. Maintenance costs were included within the acquisition costs.

Costs are in 2023 Australian dollars. US dollars were converted from AU$ to US dollars using the EPPI-Centre cost converter.

^SCIg pump cost is calculated as a weighted average of the SCIg60 Infusor and Spring Infusor pump, according to usage

Abbreviations: HM: haematological malignancies; IV: intravenous; IVIg: intravenous immunoglobulin; SCIg: subcutaneous immunoglobulin.

## Staffing Costs

The staff types and their associated wages, including 30% on-costs, are shown in Table S6 below. On-costs were estimated based on a payroll tax of 4.85%, superannuation of 11%. work cover insurance of 1.5%, annual leave of 8%, and accounting for extra leave provisions.

**Table S6. Staff type and the hourly wages**

| **Staff type** | **Weekly wage + 30% on-costs** | |
| --- | --- | --- |
|  | **AU$** | **US$** |
| Administration officer | $1,814 | $1,253 |
| Specialist consultant | $8,079 | $5,583 |
| Medical officer | $2,156 | $1,490 |
| Laboratory technician | $1,594 | $1,102 |
| Scientist | $2,281 | $1,576 |
| Senior scientist | $2,569 | $1,775 |
| Pharmacist | $3,786 | $2,616 |
| Pharmacy technician | $2,214 | $1,530 |
| Patient services assistant | $2,006 | $1,386 |
| Registrar | $3,071 | $2,122 |
| Registered nurse | $2,487 | $1,719 |
| Transfusion nurse | $2,481 | $1,715 |
| SCIg nurse | $2,841 | $1,963 |
| **On-costs items** | **% base salary** | **Source** |
| Payroll tax | 4.85% | 1. State Revenue Office Victoria |
| Superannuation^†^ | 11% | 2. Nationwide. Superannuation rates |
| Workcover insurance | 1.5% | 3. Work Safe Victoria |
| Annual leave | 8% | 4. EBA medical specialists enterprise agreement  5. EBA nurses enterprise agreement |
| Other leave provisions | 4.7% |  |

Costs are in 2023 Australian dollars. US dollars were converted from AU$ to US dollars using the EPPI-Centre cost converter.

† Superannuation is a mandatory retirement savings system that employers have to contribute to with a fixed percentage of earnings.

Abbreviations: SCIg: subcutaneous immunoglobulin

Sources: 1. <https://www.sro.vic.gov.au/historical-rates-payroll-tax-rates>; 2. <https://www.nationwidesuper.com.au/superannuation-rates-and-thresholds>; 3. <https://www.worksafe.vic.gov.au/industry-rates-and-key-dates>; 4. <https://amavic.com.au/files/amavic_medical_specialists_enterprise_agreement_2022_2026.pdf> ; 5. [www.anmfvic.asn.au/~/media/files/anmf/eba%202020/campaign%20updates/200120-NandM-EBA-master-clean.pdf](http://www.anmfvic.asn.au/~/media/files/anmf/eba%202020/campaign%20updates/200120-NandM-EBA-master-clean.pdf)

## Calculation of process costs

The cost of each process was calculated as the total of the cost of each activity within the process times the probability or likelihood of that activity occurring, incorporating both the cost of consumables and staff time. The process flowchart below and table provide an example. In this example after Activity one a decision is made either to proceed with Activity 2 or undertake Activity 3 instead.


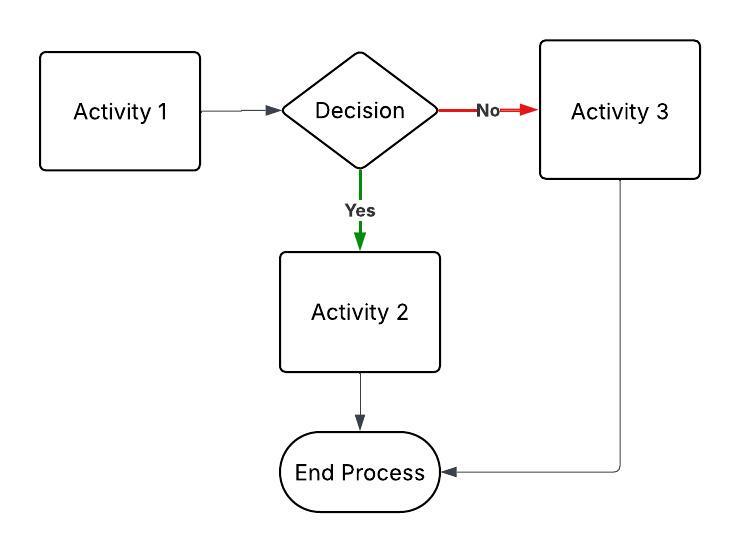


**Table S7. Total cost calculation for a process (example based on the process flowchart above)**

| **Activity** | | **prob** | **N. of consumb** | **Consumb unit cost** | **Average consumb cost ^** | **Average staff wage (per sec)** | **Average Staff time (sec)** | **Average staff Cost ^** | **Total Cost** |
| --- | --- | --- | --- | --- | --- | --- | --- | --- | --- |
| Activity 1 | | 1 | 0 | 0 | 0 | $0.0028 | 6.67 | $0.02 | $0.02 |
| Decision | Activity 2 | 0.1 | 1 | $13 | 1.3^ | $0.0028 | 7.48 | $0.02 | $1.32 |
|  | Activity 3 | 0.9 | 0 | 0 | 0 | $0.0028 | 3.95 | $0.01 | $0.01 |
| Total process cost | | | | | | | | | $1.35 |
| Total staff time (sec) | | | | | | | | | 18.1 |

^ Average consumable cost and average staff costs represent the contribution of each activity to the process costs

Consumb: consumables; prob: probability; sec: seconds

The average cost of consumables contributing to a process was calculated by multiplying the consumables unit cost by the number of consumables used and probability of that activity occurring.

$$\bar{C}=\sum_{i=1}^{n} p_{i}\cdot q_{i}\cdot c_{i}$$

$$\bar{C}=\text{Average cost of consumables across an activity}$$

$$p_{i}=\text{Probability of activity }i\text{ occurring}$$

$$q_{i}=\text{Quantity of consumables used for activity }i$$

$$c_{i}=\text{Unit cost of the consumable for activity }i$$

The average staff cost contributing to a process is calculated by multiplying the average staff wage, average staff time and the probability of that activity occurring.

$$\bar{S}=\sum_{i=1}^{n} p_{i}\cdot t_{i}\cdot w_{i}$$

$$\bar{S}=\text{Average staff cost across all activities}$$

$$p_{i}=\text{Probability of activity }i\text{ occurring}$$

$$t_{i}=\text{Average staff time for activity }i$$

$$w_{i}=\text{Average staff wage rate for activity }i$$

The total process cost is the sum of the average staff cost and the average consumable cost for all activities contributing to a process.

$$T=\sum_{i=1}^{n} \left( \bar{S_{i}}+\bar{C_{i}} \right)$$

$$T=\text{Total process cost across all activities}$$

$$\bar{S_{i}}=\text{Average staff cost contributed to the process by activity }i$$

$$\bar{C_{i}}=\text{Average consumable cost contributed to the process for activity }i$$

The cost of equipment (when equipment was used) was added to the total process costs, as it had already been calculated as cost per episode.

## Calculation of total annual costs per patient

The calculation of total annual costs per patient was separated for new and continuing patients following the process flow in the master flowchart for IVIg and SCIg.

The cost per episode per patient was calculated separately for separate branches within the IVIg and SCIg master flowcharts by adding up the cost of the processes included in each branch according the probabilities in the flowchart.

The total branch cost is the sum of process cost times the probability of each process occurring

$$B_{c}=\sum_{i=1}^{n} P_{i}\times T_{i}$$

$$B_{c}=\text{Total branch cost}$$

$$P_{i}=\text{Probability of process }i\text{ occurring within that branch}$$

$$T_{i}=\text{Total cost of process }i$$

The following branches were identified in the IVIg flowchart (Table M2): (A) processes occurring only once (only applied to new patients to IVIg), after this (B) IVIg was given every month, except for(C) patients attending 6-monthly reviews.

**Table S8. Processes in IVIg master flowchart branches by number of times repeated within a year**

| 1. Only once for new patients | 1. Every month | 1. Every 6 months |
| --- | --- | --- |
| 9a New IVIg authorisation | 1. IVIg lab dispensing | 9e IVIg review |
| 1. IVIg lab dispensing | 10. IVIg MIU paperwork | 9a New IVIg authorisation |
| 10. IVIg MIU paperwork | 3a. Individual issue of IVIg | 1. IVIg lab dispensing |
| 3a. Individual issue of IVIg | 3b. Multiple issue of IVIg | 10. IVIg MIU paperwork |
| 3b. Multiple issue of IVIg | 13. Transport of IVIg | 3a. Individual issue of IVIg |
| 13. Transport of IVIg | 6a. IVIg patient preparation | 3b. Multiple issue of IVIg |
| 6a. IVIg patient preparation | 6b IVIg infusion | 13. Transport of IVIg |
| 6b IVIg infusion | 11. Infusion reaction investigation | 6a. IVIg patient preparation |
| 11. Infusion reaction investigation |  | 6b IVIg infusion |
|  |  | 11. Infusion reaction investigation |

Abbreviations: IVIg: intravenous immunoglobulin; MIU: Medical Infusion Unit

The total branch cost (Bc) was calculated for each branch using the formula above and represented one IVIg episode. The model assumptions included 13 IVIg episodes per year (one IVIg episode every 4 weeks) for both new and continuing patients. Therefore, the annual administration cost of IVIg was calculated as 13 infusion episodes across the following branches:

$$\text{Total Annual Administration Cost (new IVIg patient)}=1\times Bc(A)+11\times Bc(B) 1\times Bc(C)$$

$$\text{Total Annual Administration Cost (continuing IVIg patient)}=11\times Bc(B) + 2\times Bc(C)$$

The following branches were identified in the SCIg flowchart (Table M3) and the total cost per episode was calculated for each of them: (A) processes occurring “Only once” (only applied to new patients to SCIg), after this (B) SCIg was given every 8 weeks, except for(C) patients attending 6-monthly reviews.

**Table S9. Processes in SCIg master flowchart branches by number of times repeated within a year**

| 1. Only once for new patients | 1. Every 8 weeks | 1. Every 6 months |
| --- | --- | --- |
| 7a. New SCIg patient enrolment | 8a. Patient requires more SCIg | 9d Reviewing outcomes and continue authorisation for SCIg |
| 9b. New SCIg patient authorisation | 9c. New script for continuing SCIg patient | 9f. Existing SCIg patient re-authorisation |
| 4a Pharmacy preparation to dispense (new) SCIg patient | 4b. Pharmacy preparing to dispense (continuing) SCIg patient | 4b. Pharmacy preparing to dispense (continuing) SCIg patient |
| 4c. Pharmacy preparing to collect SCIg from Blood Bank | 4c. Pharmacy preparing to collect SCIg from Blood Bank | 4c. Pharmacy preparing to collect SCIg from Blood Bank |
| 2. Laboratory SCIg dispensing | 2. Laboratory SCIg dispensing | 2. Laboratory SCIg dispensing |
| 5. Laboratory SCIg issue | 5. Laboratory SCIg issue | 5. Laboratory SCIg issue |
| 4d. Pharmacy SCIg dispensing | 4d. Pharmacy SCIg dispensing | 4d. Pharmacy SCIg dispensing |
| 7b. SCIg training preparation |  |  |
| 7c. First SCIg training |  |  |
| 7d. Second SCIg training |  |  |
| 8d. SCIg Administration Training and Competency Report |  |  |

Abbreviations: SCIg: subcutaneous immunoglobulin

In all of the branches, SCIg patients are provided with an 8-week supply of product and consumables; therefore, to calculate the weekly cost per episode each of the branch costs was divided by 8. The model assumptions included 52 SCIg episodes per year (one SCIg episode every week) for both new and continuing patients. Therefore, the annual administration cost of SCIg was calculated as 52 infusion episodes across the following branches:

$$\text{Total Annual Administration Cost (new SCIg patient)}=8\times Bc(A)+36\times Bc(B) + 8\times Bc(C)$$

$$\text{Total Annual Administration Cost (continuing SCIg patient)}=36\times Bc\left( B \right)+16\times Bc\left( C \right)$$

For both IVIg and SCIg patients, regular processes that were not included in the master flowchart (i.e., blood fridge checks, Ig order and delivery) and fixed costs (salary overheads for administrative and oversight duties) were added to the total annual cost.

The cost of IVIg and SCIg product per episode was multiplied by 13 and 52, respectively, and added to the total annual administration costs to obtain the total annual costs in the base case model. The cost of the take-home pump for new SCIg patients was also added to the new SCIg patient total annual costs.

# Bootstrapping and 95% CI

We used bootstrapping with 1000 iterations to estimate the 95% confidence intervals around the total annual costs and cost difference between IVIg and SCIg, considering the uncertainty associated with the probabilities in the model. We used a proportion-based approach to estimate the 95% CI margin of error for the probabilities in the flowcharts and from this calculated a lower and upper limit. The following formulas were used:

$$Margin of error=1.96 x \sqrt{\frac{p(1-p)}{n}}$$

*p=* probability

*n=* sample size (number of episodes)

Lower and upper limits were calculating in Excel using:

*Lower limit = MAX(0, p - margin of error)*

*Upper limit = MIN(1, p + margin of error)*

Stata 18 was used to calculate 1000 iterations of the probabilities drawing independently from a uniform distribution between the lower and upper limit for each probability. The 95% CI were reported out of these 1000 iterations.

# Individual process flowcharts

## **Laboratory IVIg dispensing**


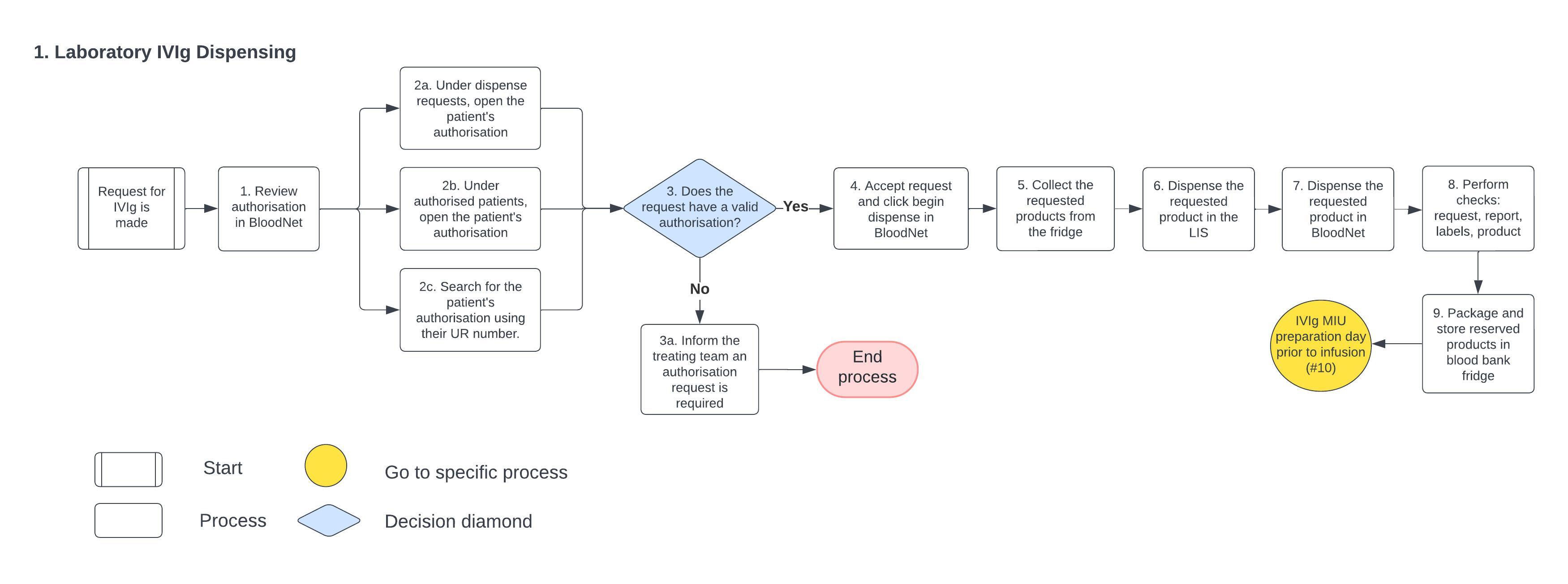


1. Laboratory SCIg dispensing


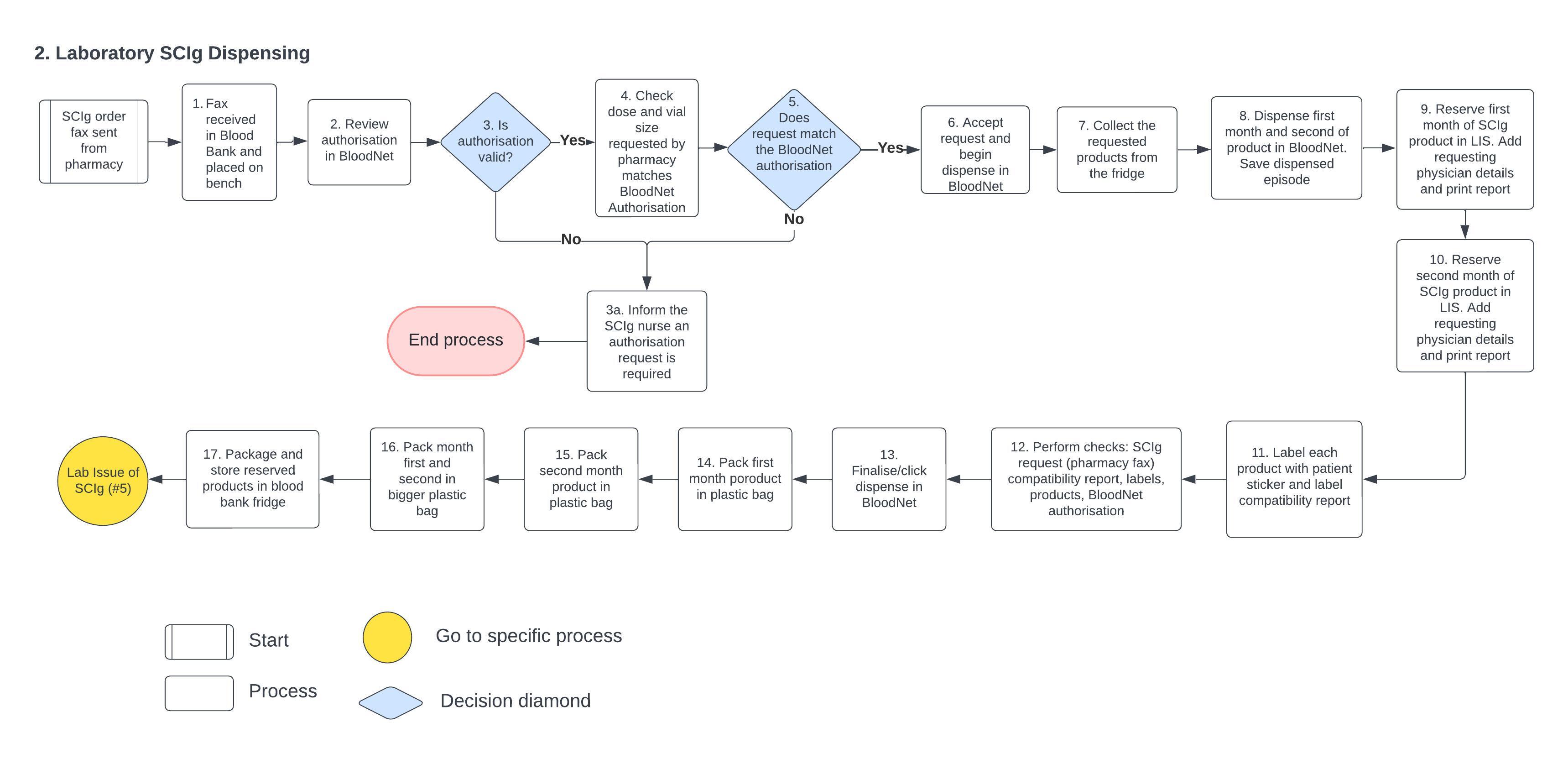


## Laboratory IVIg issue: 3a. Individual issue. 3b. Multiple issue


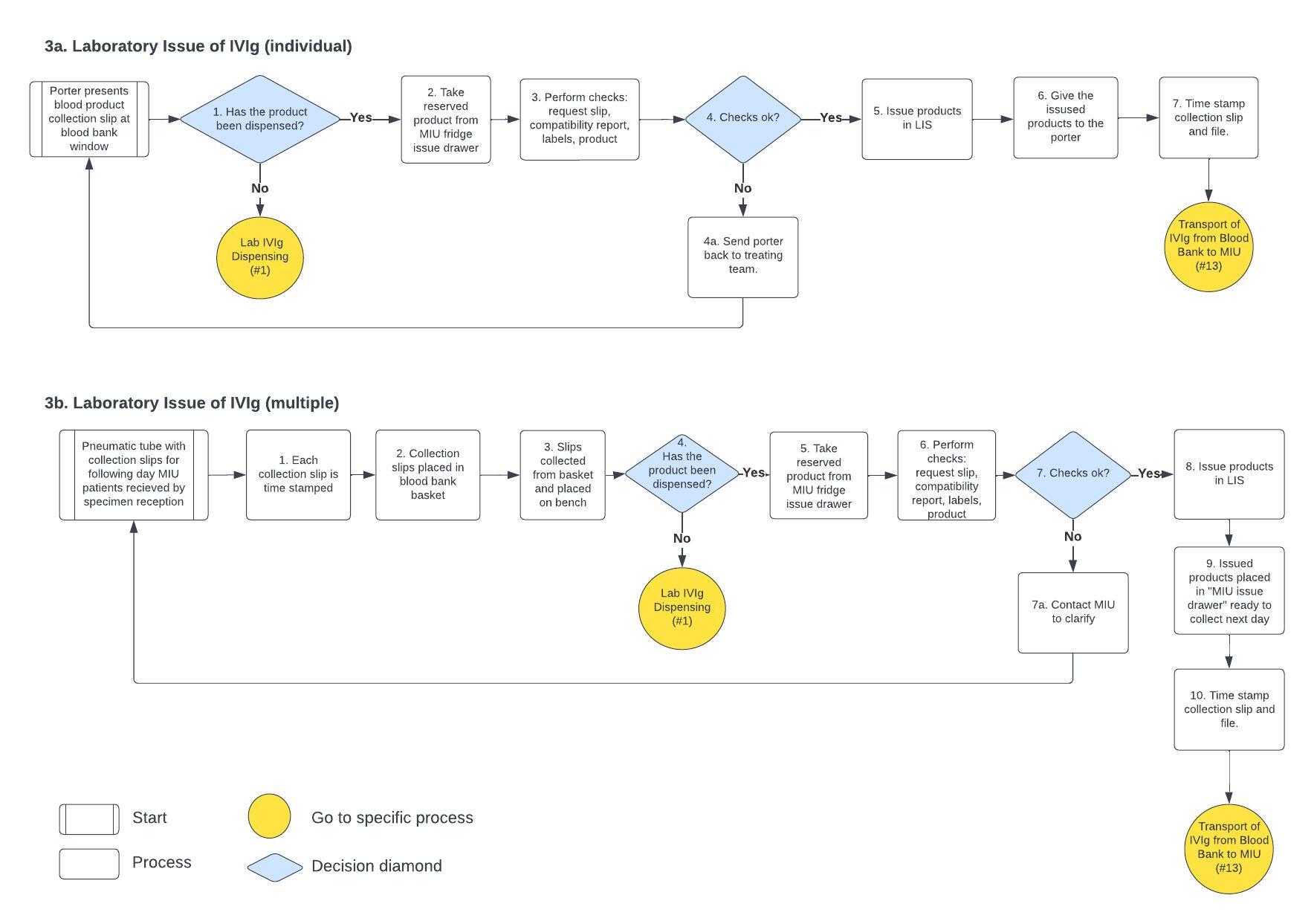


## Pharmacy SCIg dispensing: 4a. Pharmacy preparing to dispense (new) SCIg patient. 4b. Pharmacy preparing to dispense (continuing) SCIg patient. 4c. Pharmacy preparing to collect SCIg from Blood Bank. 4d. Pharmacy SCIg dispensing.


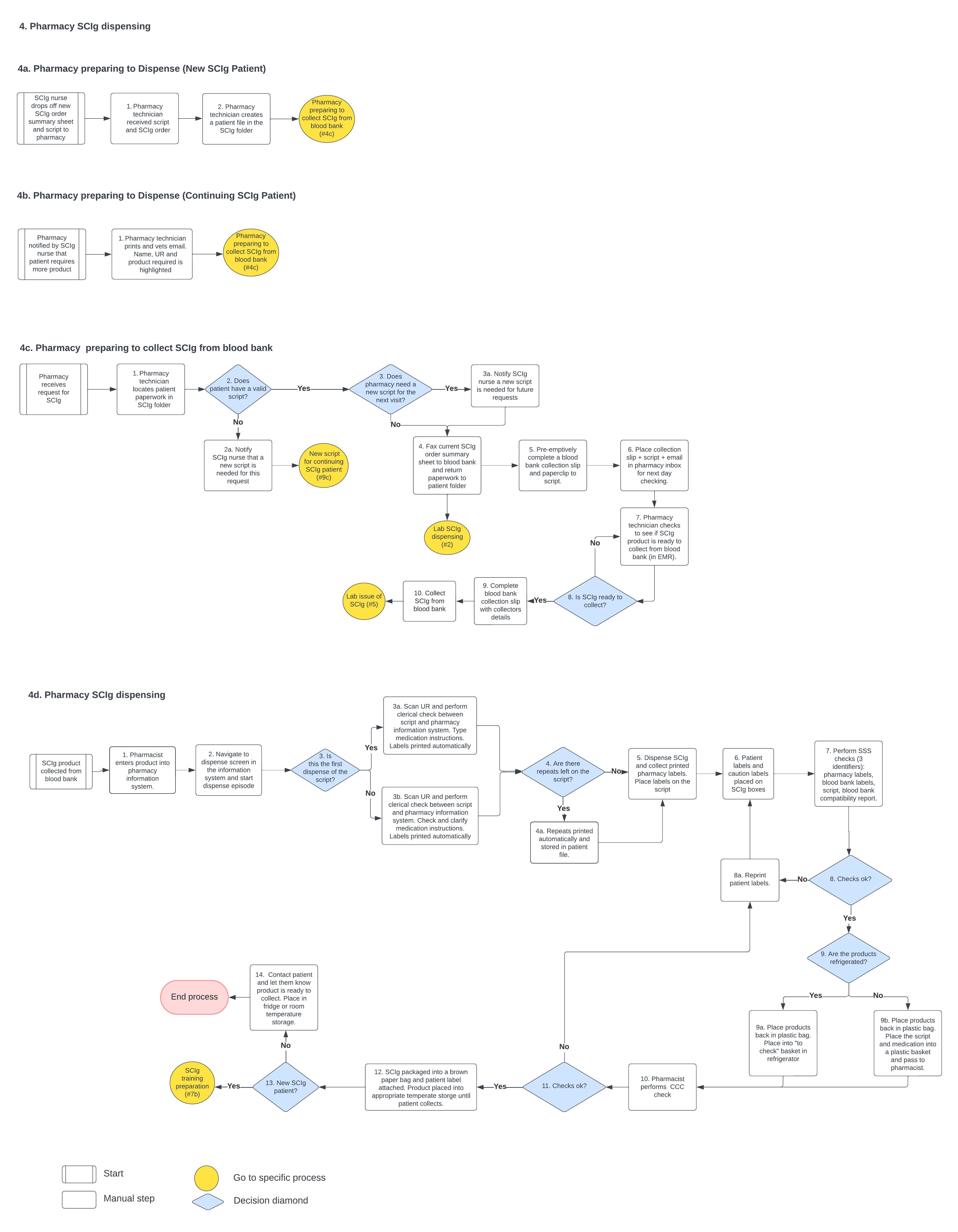


## Laboratory SCIg issue


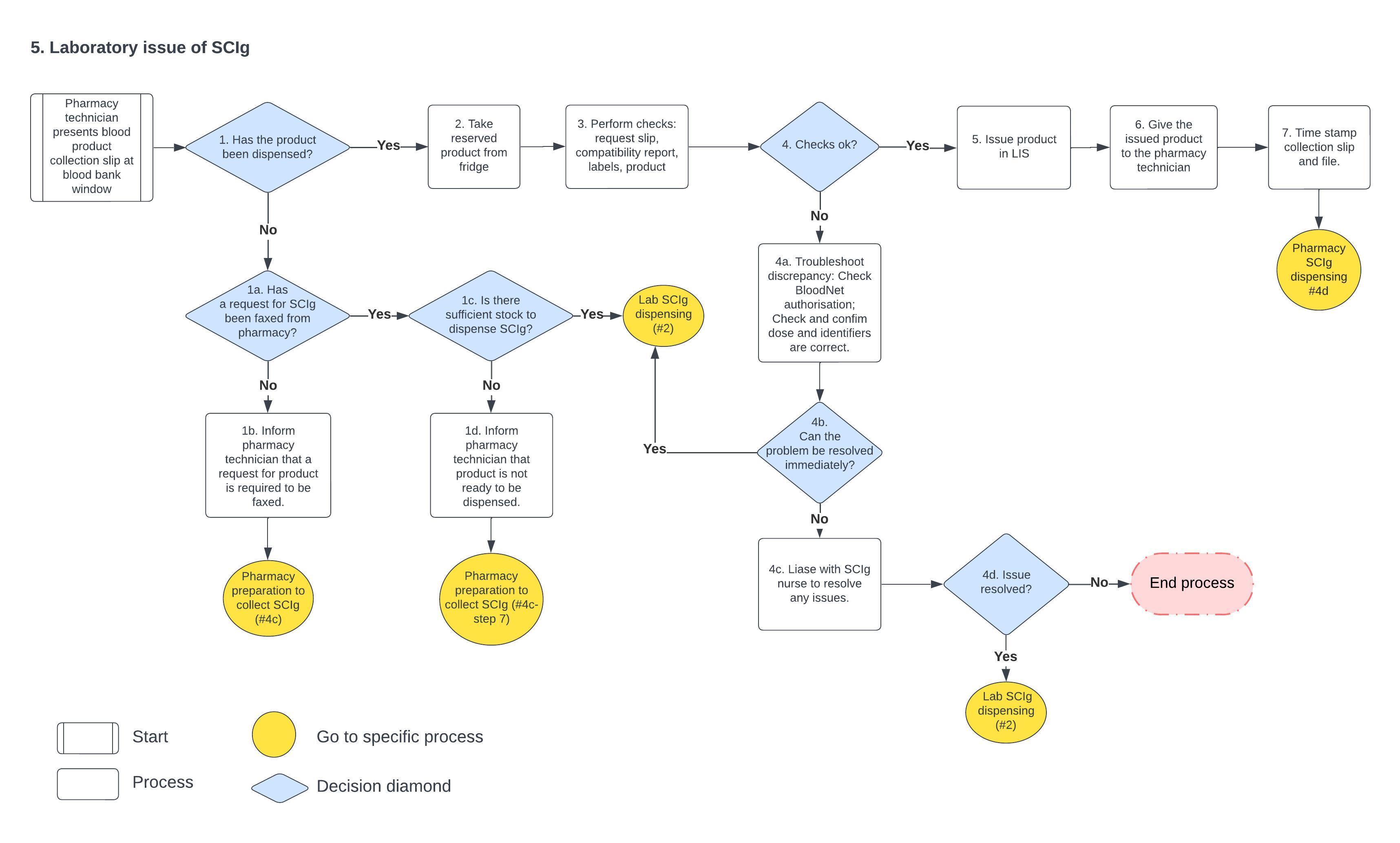


## Admin of IVIg: 6a. IVIg patient preparation / 6b. IVIg infusion


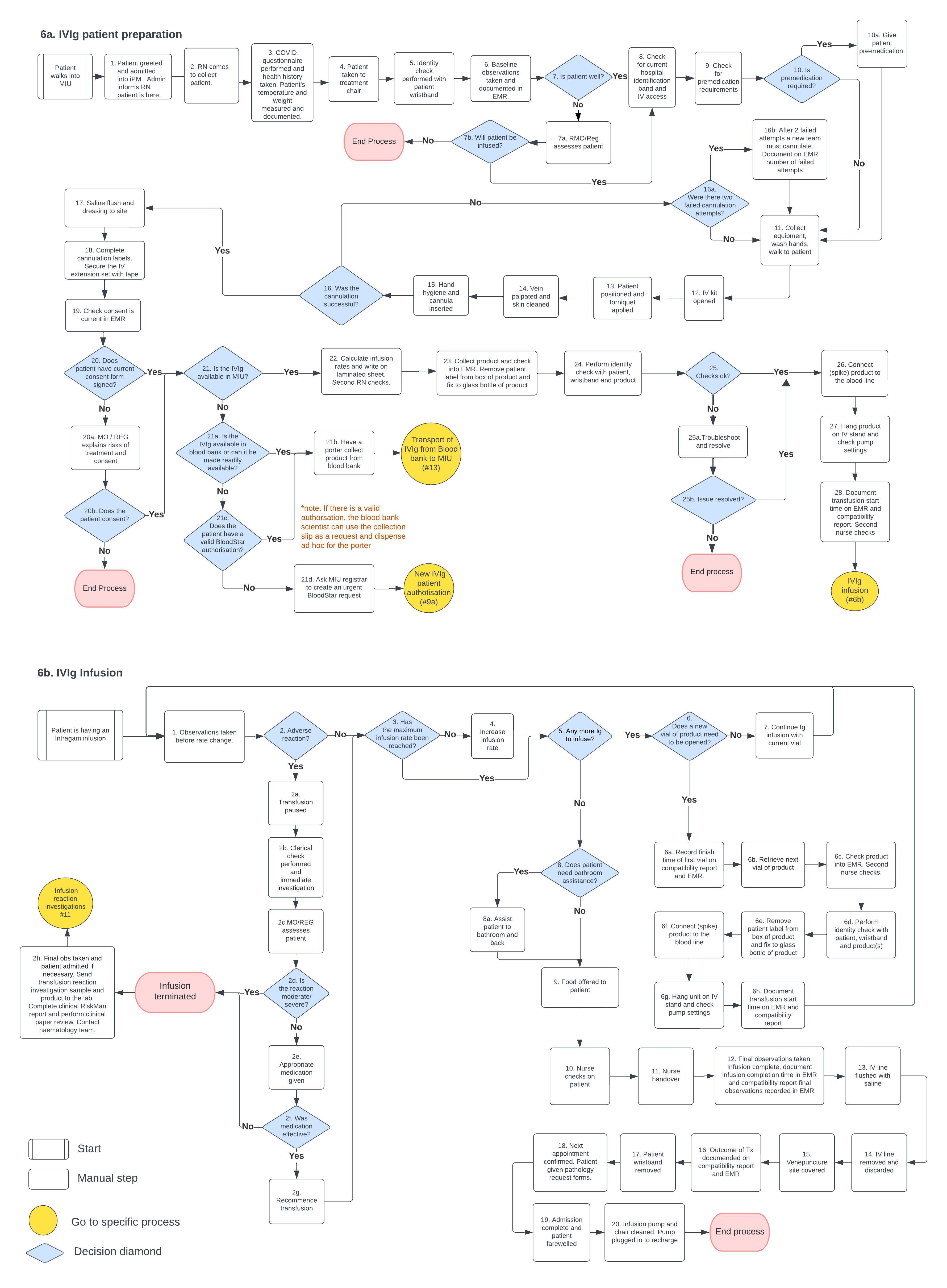


## 7a. New SCIg patient enrolment. 7b. SCIg training preparation. 7c. First SCIg training. 7d. Second SCIg training


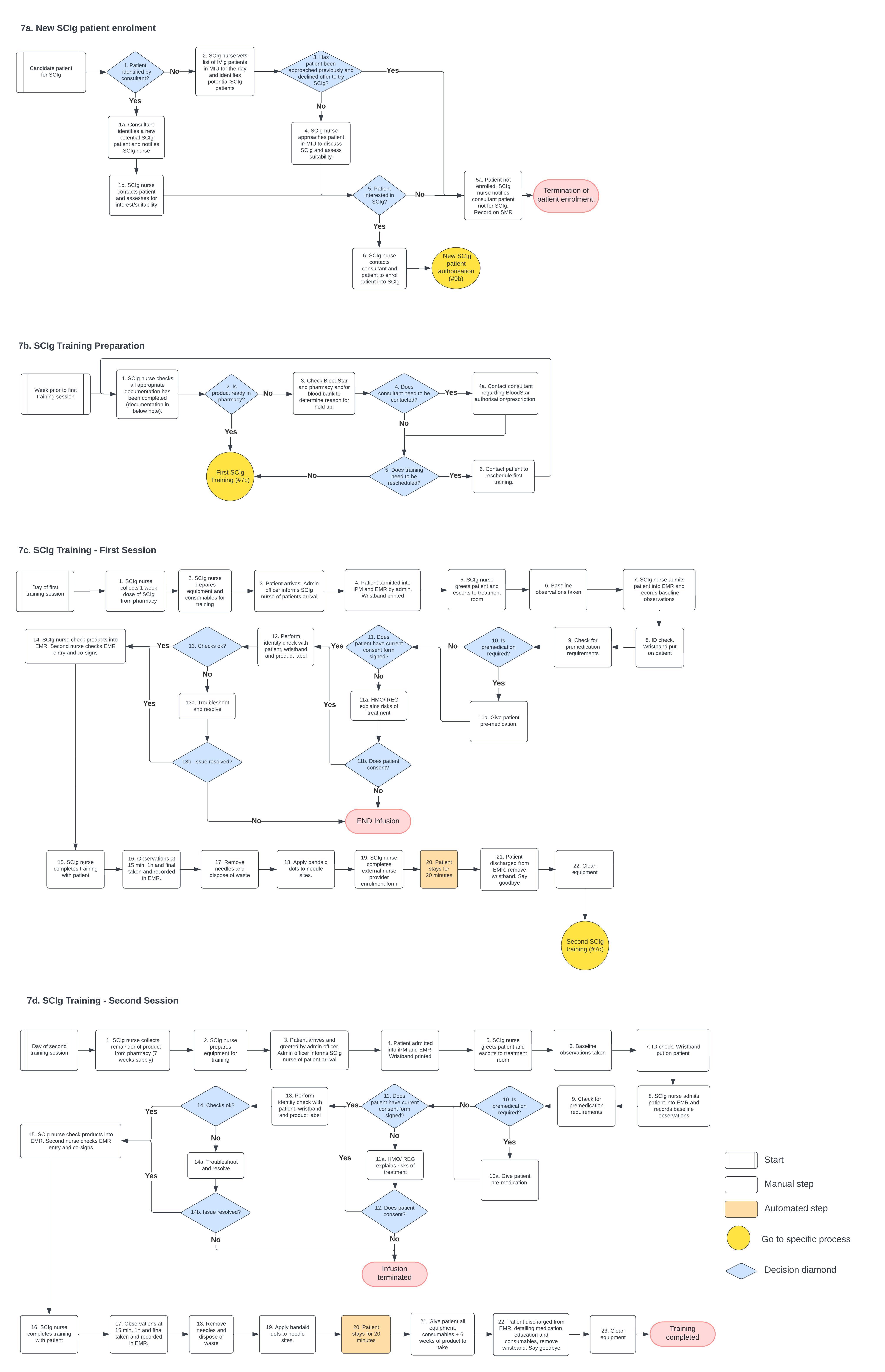


## 8a. Patient requires more SCIg. 8b/8c. Patient requires more consumables. 8d. SCIg Administration Training and Competency Report


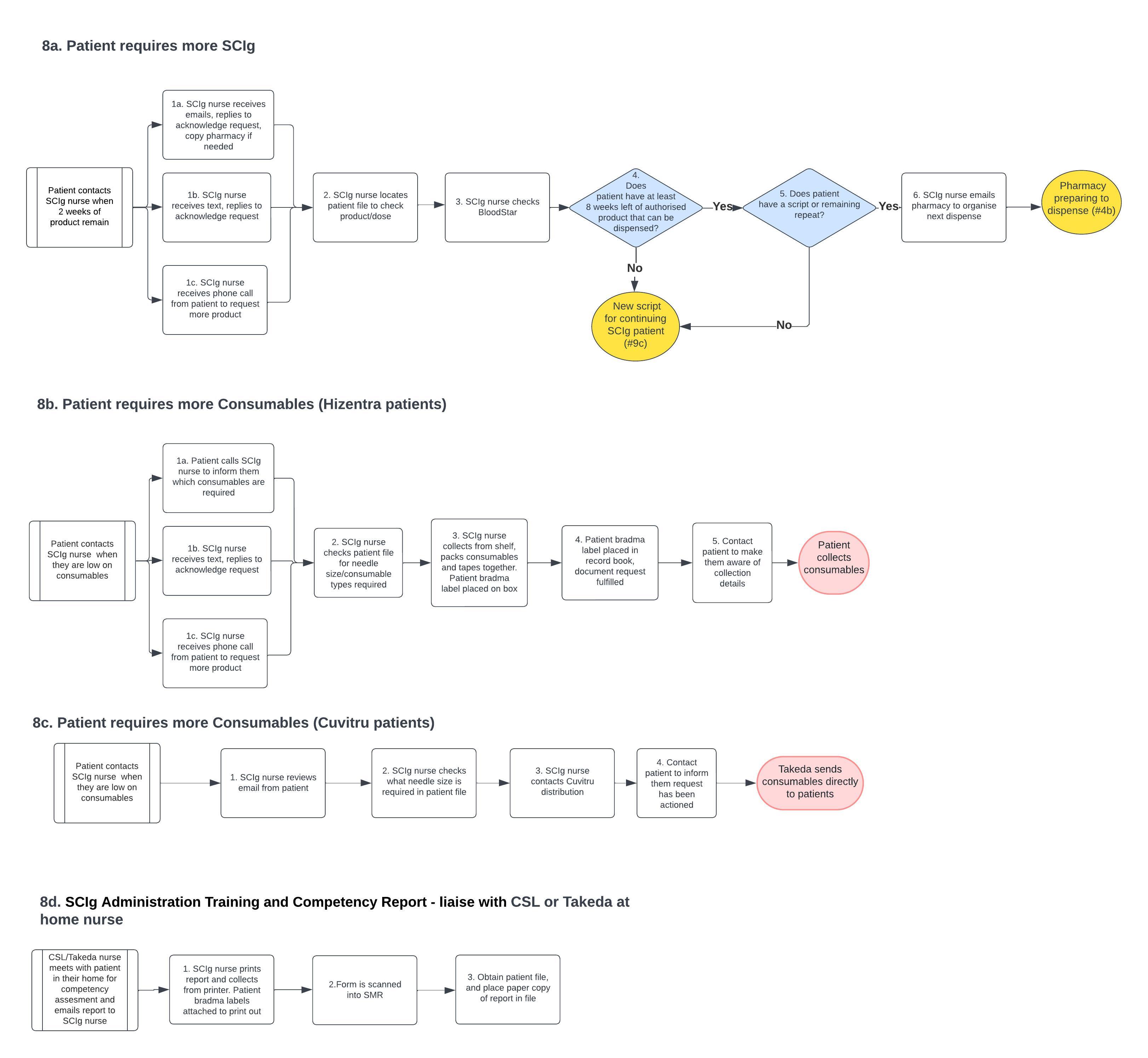


## 9a. New IVIg patient authorisation. 9e Reviewing outcomes and continue authorisation for IVIg


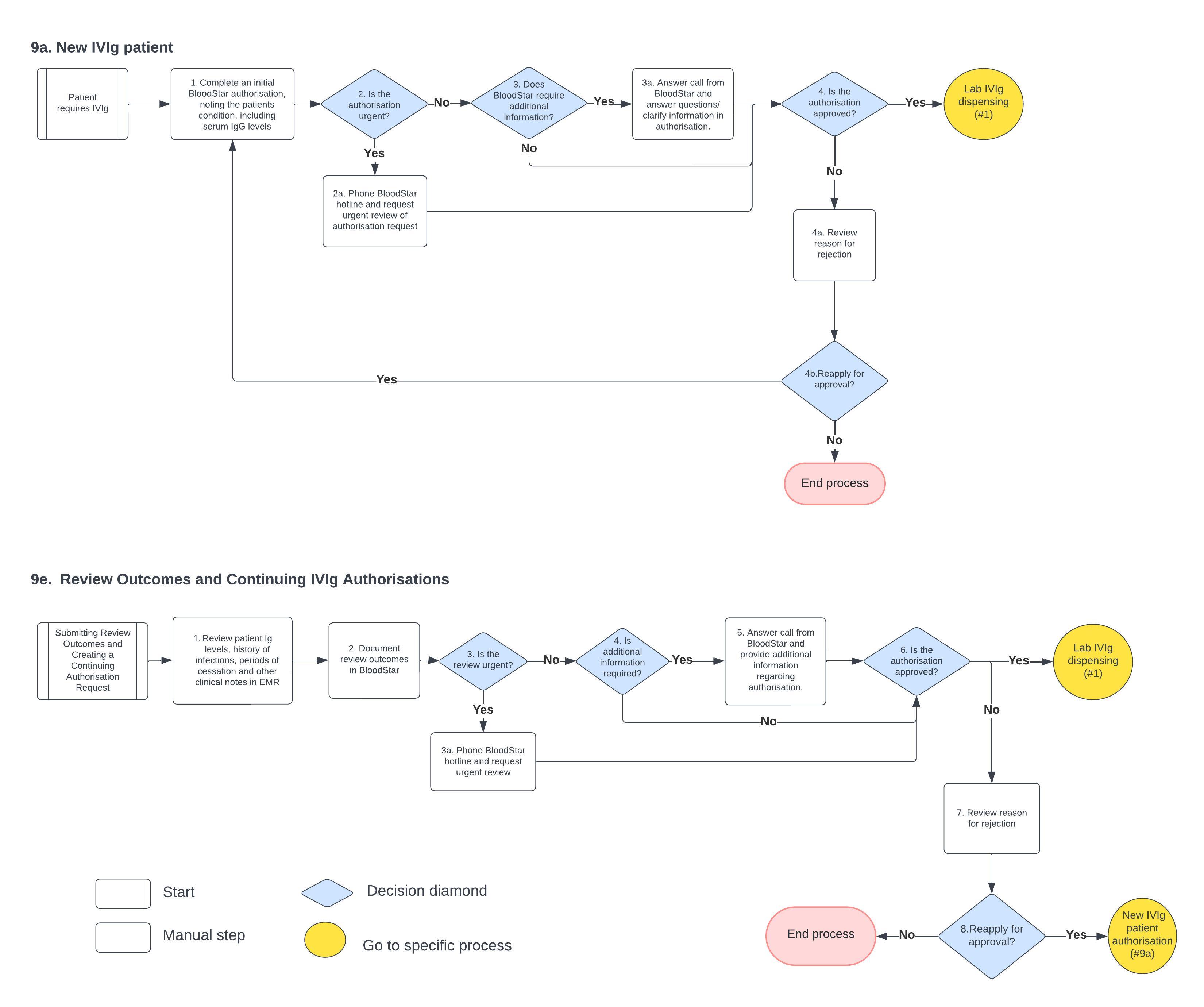


## 9. 9b. New SCIg patient authorisation. 9c. New script for existing SCIg patient. 9d Reviewing outcomes and continue authorisation for SCIg. 9f. Existing SCIg patient re-authorisation


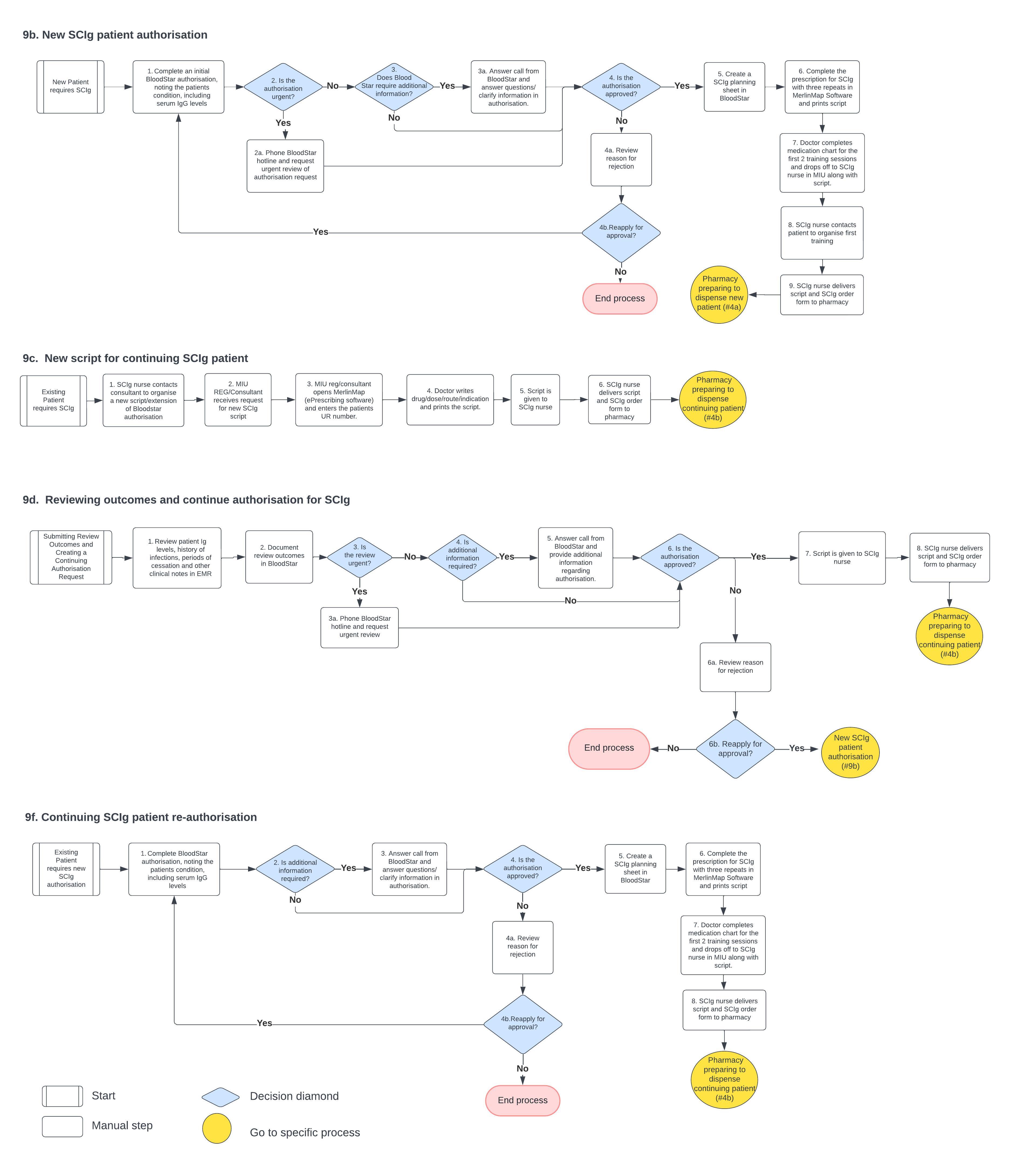


## IVIg MIU preparation day prior to infusion


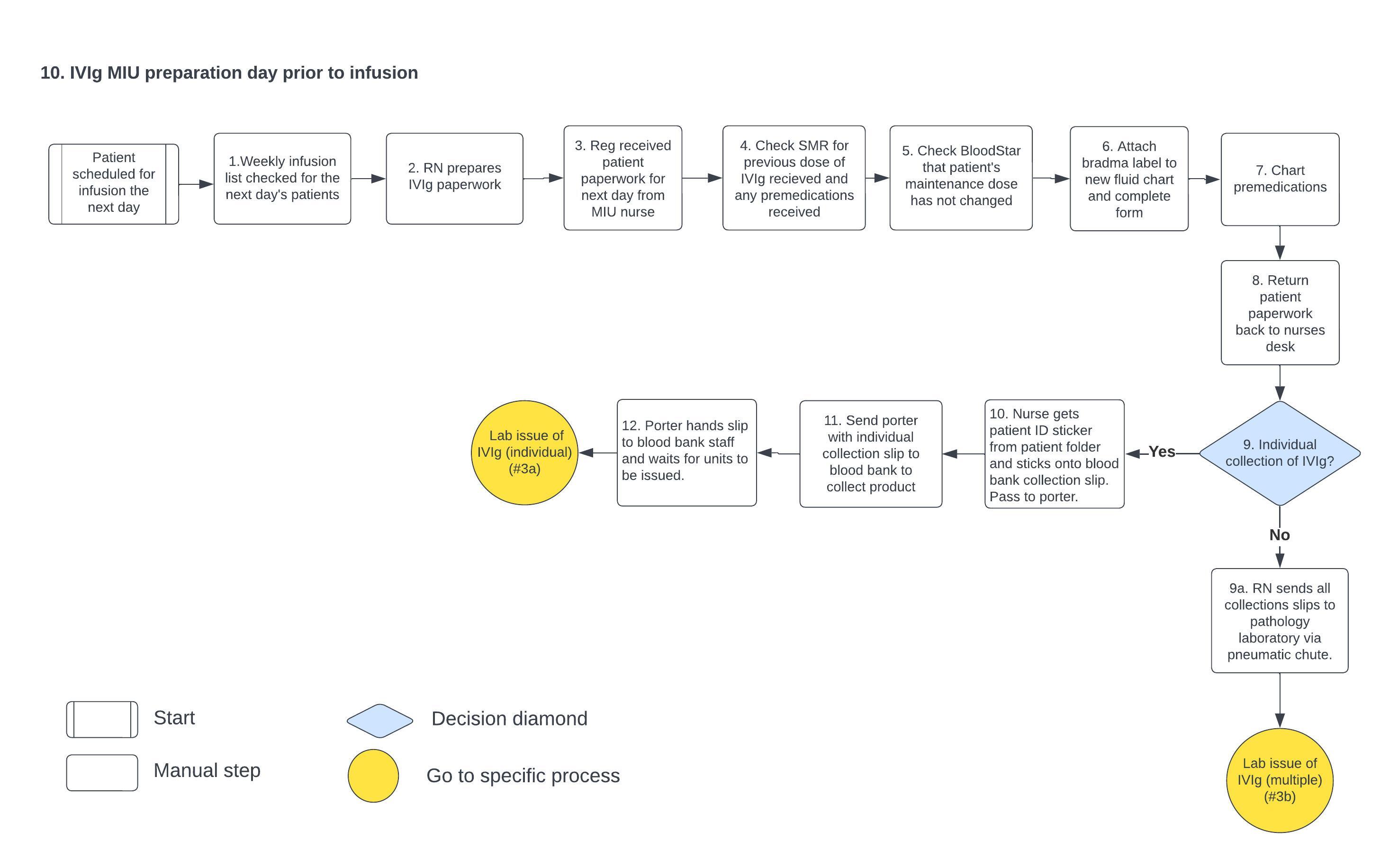


## Infusion reaction investigation: 11a. Laboratory testing. 11b. Elution. 11c. Analysis and notification


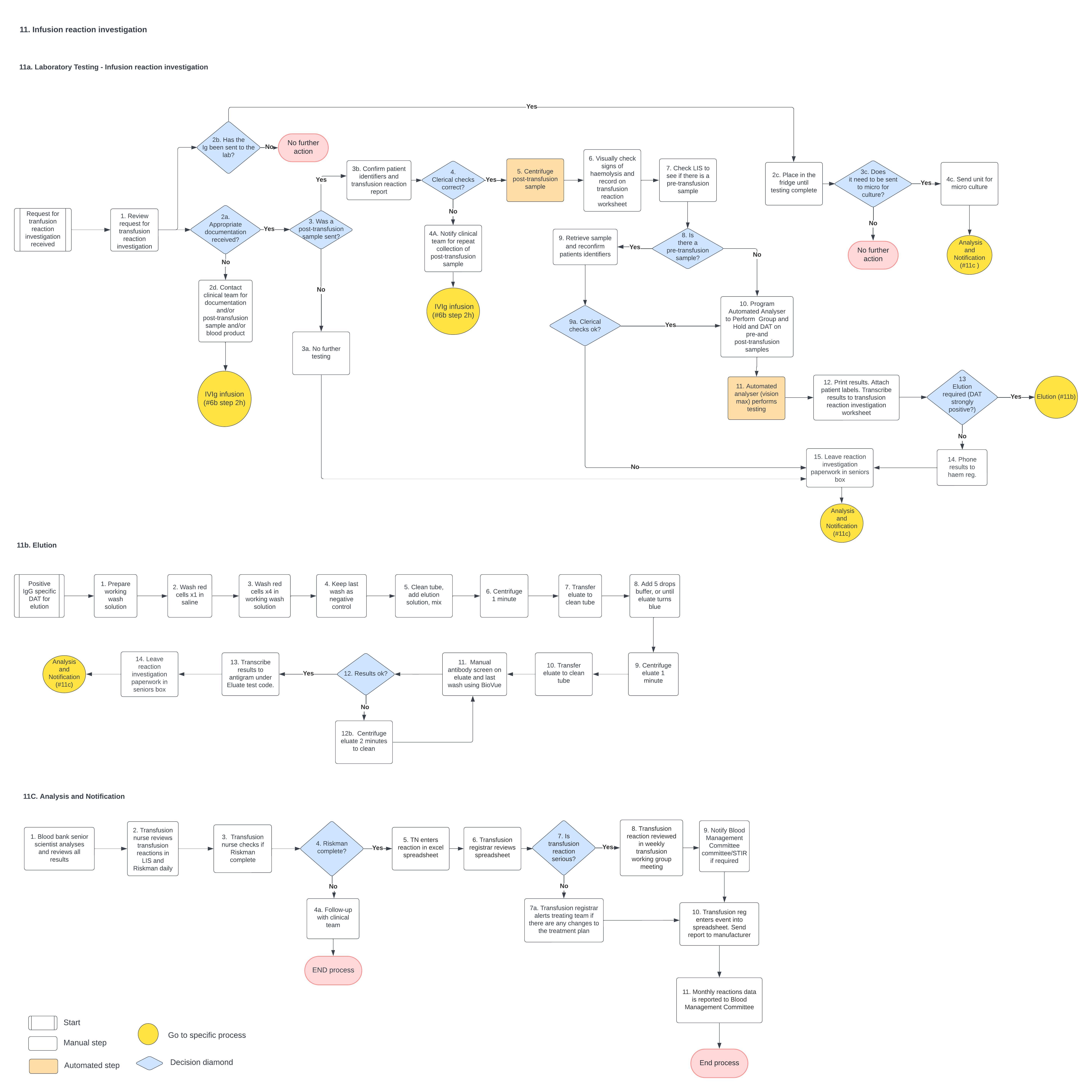


## Blood fridge checks and maintenance


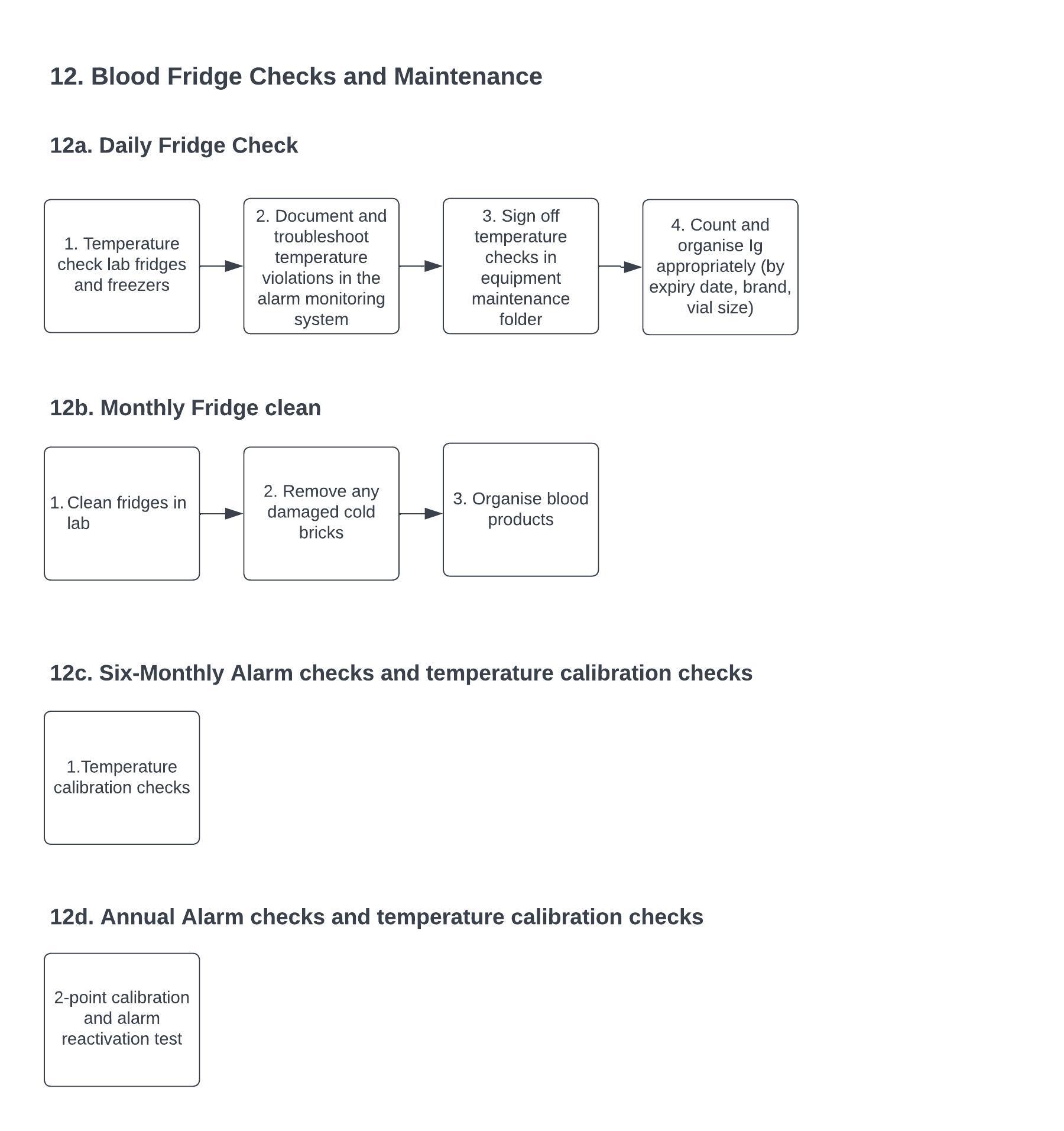


## 13. Transport of IVIg from Blood Bank to MIU


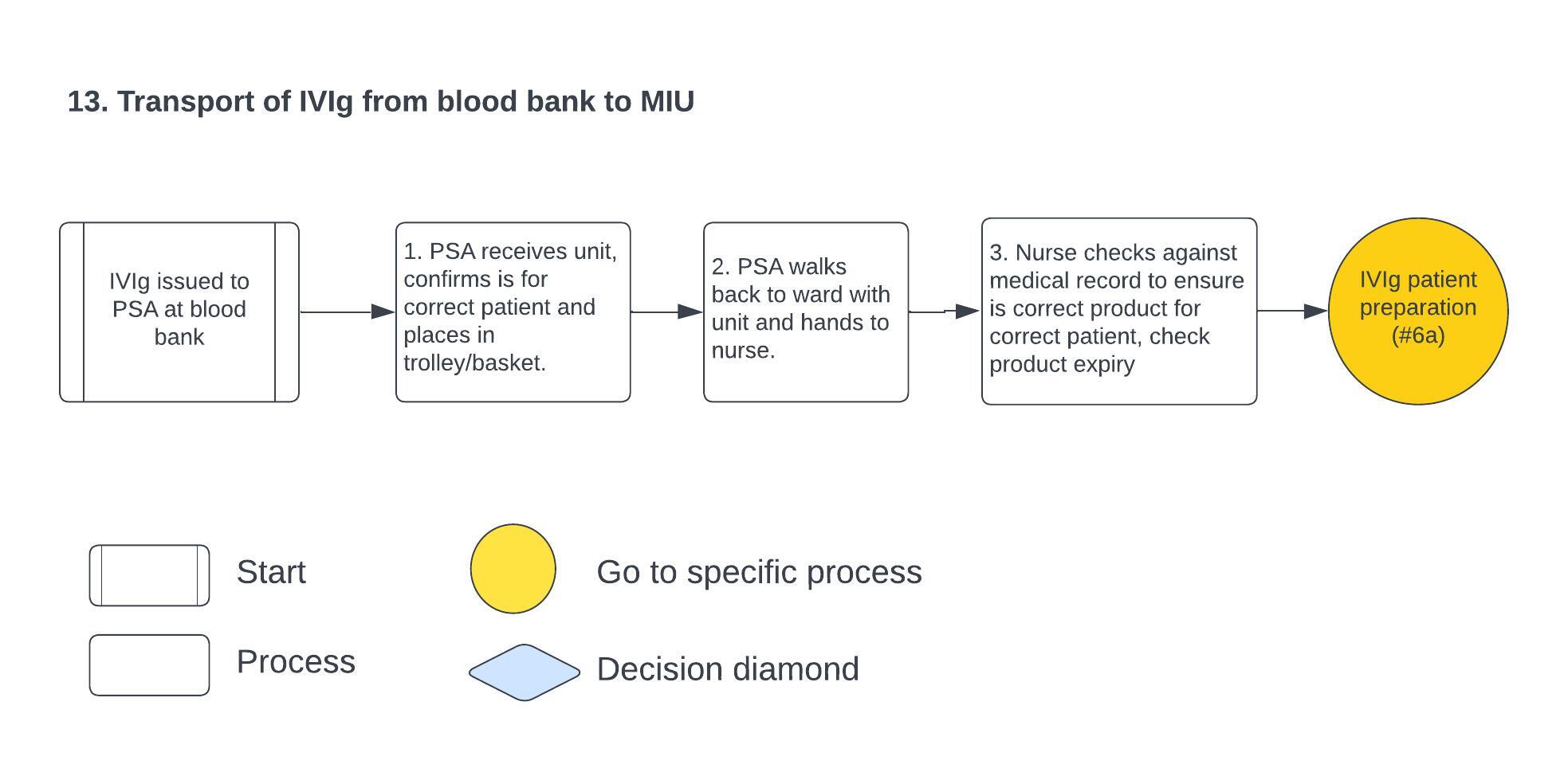


## . Ig order and delivery: 14a. Daily routine order of Ig. 14b. Daily Ig delivery


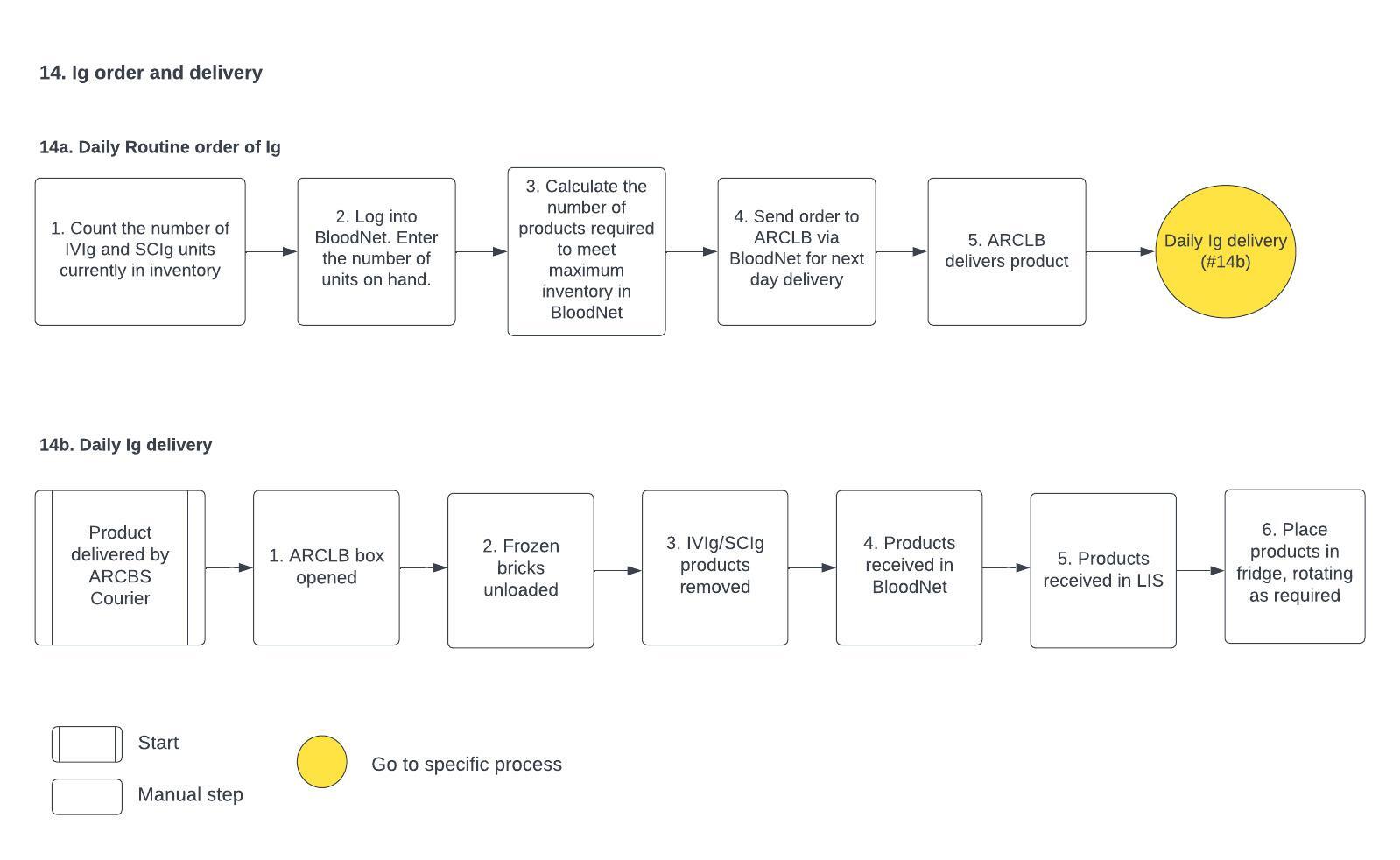

Supplement: Supplementary file 1 — (DOCX 6.29 MB) [file 520_2026_10551_MOESM1_ESM.docx]
